# Benznidazole in vitro dissolution release from a pH-sensitive drug delivery system using Zif-8 as a carrier

**DOI:** 10.1007/s10856-021-06530-w

**Published:** 2021-05-17

**Authors:** Leslie Raphael de Moura Ferraz, Alinne Élida Gonçalves Alves Tabosa, Débora Dolores Souza da Silva Nascimento, Aline Silva Ferreira, José Yago Rodrigues Silva, Severino Alves Junior, Larissa Araújo Rolim, Pedro Jose Rolim-Neto

**Affiliations:** 1 Laboratório de Tecnologia dos Medicamentos (LTM), Department of Pharmaceutical Sciences, Federal University of Pernambuco, Av. Prof. Arthur de Sá, s/n, Cidade Universitária, 50740-521 Recife, PE Brazil; 2 Laboratório de Terras Raras (BSTR), Fundamental Departament of Chemistry, Federal University of Pernambuco, Av. Jornalista Aníbal Fernandes, s/n - Cidade Universitária, 50740-560 Recife, PE Brazil; 3 Central Analítica de Fármaco, Medicamentos e Alimentos (CAFMA), Federal University of Vale do São Francisco, Av. José de Sá Maniçoba, s/n, Centro, 56304-917 Petrolina, PE Brazil

## Abstract

Chagas disease is a neglected tropical disease caused by the flagellate protozoan *Trypanosoma cruzi* (*T. cruzi*). Endemic in underdeveloped and developed countries, due to the migratory movement, it is considered a serious public health problem. Endemic in underdeveloped countries and due to the migratory movement, in developed countries as well, it is considered a serious public health problem. One of the reasons for this is a weak therapeutic arsenal, represented only by the drug benznidazole (BNZ) which, although it promotes significant cure rates in the acute phase of the disease, presents serious problems of toxicity and bioavailability, mainly due to its low aqueous solubility. Several studies have presented several drug delivery systems (DDS) based on BNZ aiming at enhancing its solubility in aqueous medium and, with this, promoting an increase in the dissolution rate and, consequently, in its bioavailability. However, the present work is a pioneer in using a zeolitic imidazolate framework as a carrier agent for a DDS in order to promote a pH-sensitive modulation of the drug. Thus, this work aimed to develop a novel DDS based on BNZ and the ZIF-8 to use it in development of prolonged-release dosage forms to alternative treatment of Chagas disease. The BNZ@ZIF-8 system was obtained through an ex situ method selected due to its higher incorporation efficiency (38%). Different characterization techniques corroborated the obtainment and drug release data were analyzed by in vitro dissolution assay under sink and non-sink conditions and setting the kinetic results through both model dependent and independent methods. Under sink conditions, at pH 4.5, BNZ and BNZ@ZIF-8 showed similar release profile, but the DDS was effective in promoting a prolonged release. At pH 7.6, after 7 h, BNZ showed a lower release than BNZ@ZIF-8. On the other hand, in non-sink conditions at pH 4.5 the BNZ presented 80% of drug release in 3 h, while the DDS in 6 h. At pH 7.6, BNZ presented a release of 80% in 2 h, while the DDS reaches it in only at 12 h. Therefore, at pH 4.5 the DDS BNZ@ZIF-8 showed a faster release with a burst effect, while at pH 7.6 it showed a prolonged and controlled release. Finally, it is evident that a promising DDS pH-sensitive was obtained as a novel carrier that might be able to prolongs BNZ release in dosage forms intended for the alternative treatment of Chagas disease.

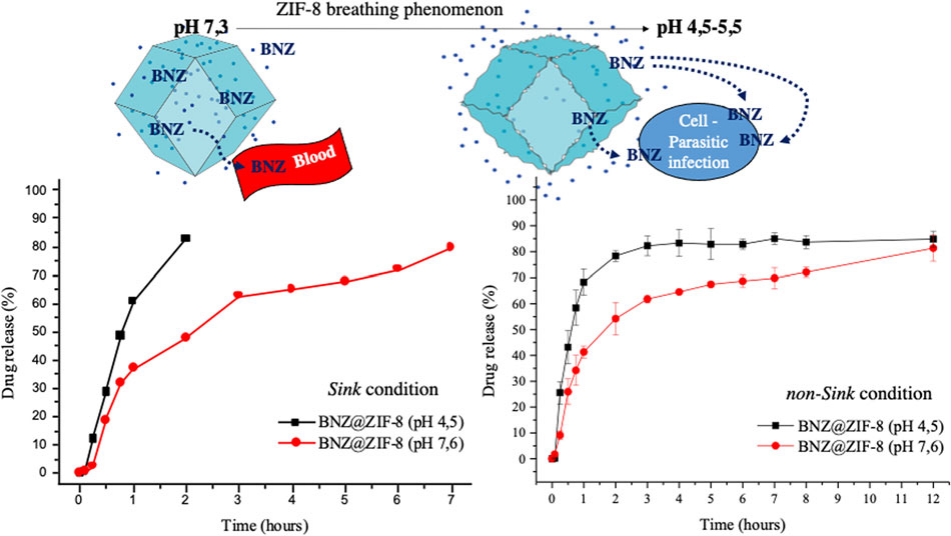

## Introduction

Chagas disease, also known as American trypanosomiasis, is a neglected tropical disease endemic in 27 countries of the American continent. It is estimated that 65 million people on the American continent live in exposure areas and are at risk of contracting the disease. Potentially fatal, the disease is caused by the parasite protozoan *Trypanosoma cruzi (T. cruzi)*, and among neglected diseases, is the parasitic infection with the greatest socioeconomic impact in Latin America, being responsible for an annual loss of about US $1.2 billion, mainly due to the loss of productivity [1–5].

Benznidazole (BNZ) (N-benzyl-2-nitroimidazole-1-acetamide) is a broad spectrum nitroimidazole derivative of antiprotozoal and antibacterial pharmacological activity, and it is the first-choice drug and the only one in the therapeutic arsenal against the disease. However, BNZ is far from ideal. The drug may cause toxic reactions and undesirable side effects like metronidazole, such as nausea, headache, anorexia, abdominal pain, weight loss, dizziness, asthenia, vomiting, rash, and ataxia [6–8].

One of the major problems associated with BNZ is the complex dosing regimens that make treatment resistance a major problem. Other relevant disadvantages are the large doses administered, prolonged treatments, as well as the high incidence of side effect reactions, which are probably related to the low solubility of the drug. BNZ is classified as a class II drug (reduced solubility and high permeability) according to the biopharmaceutical classification system (BCS), being poorly soluble in water and aqueous fluids, besides having limited absorption by the rate of dissolution and solubility. There is, therefore, considerable interest in the development of strategies to improve the release and solubility of BNZ and, possibly, to thereby increase the patient’s compliance [9, 10].

It is in this context of therapeutic optimization that the development of Drug Delivery Systems (DDS) for modulated drug release is inserted. DDS provides some advantages over conventional dosage forms because of their improved safety, efficacy, and reduced toxicity, resulting in a lower incidence of undesirable effects and increased patient compliance and convenience by keeping the drug concentration constant for a prolonged period using a single dosage. In these systems, the drug is bound to a carrier, which is responsible for improve the limiting physicochemical drug properties, being particularly interesting for BCS class II drugs [11, 12].

The Zeolitic Imidazolate Framework (ZIF) is a subclass of Metal Organic Framework (MOF) and therefore has interesting characteristics such as microporosity, high surface area, and kinetic stability, besides high thermal stability [13, 14]. However, recent innovations in the field of materials science have given ZIFs even more advantages over other MOFs. Although other BNZ-based DDS were mentioned, the system proposed by the present work is innovative due to the inherent characteristic of the ZIF framework in dissociating into Acidic pH, which promotes pH-sensitive release modulation. In this context, one particular ZIF can be highlighted, ZIF-8, which has its structure stabilized due to the bridges between the 2-methylimidazole and the tetrahedral zinc, often obtaining success as drug carriers [15–18].

ZIF-8 acts as an intelligent excipient capable of promoting the association and intercalation of active pharmaceutical ingredient (API) in the space between adjacent lamellae or through the entire extension of a single lamella. In addition, ZIF-8 have good biocompatibility, likely because zinc is the second most abundant metal in the human body and the 2 methyl imidazole scaffold is found in the amino acid histidine [16–20]. Therefore, the present work aims to develop and evaluate the in vitro dissolution release of benznidazole from a novel DDS using ZIF-8 as a carrier, as an initial stage of the preformulation study of solid dosage forms intended for the alternative treatment of Chagas disease.

## Materials and methods

### Materials

The BNZ was donated from the Laboratório Farmacêutico de Pernambuco (LAFEPE®), lot 301045 (99.5%), while the ZIF-8, lot S45328-308, was donated by the Laboratório de Terras Raras of Federal University of Pernambuco (UFPE). Acetone (Modern Chemistry®, lot 03196) and ultra-pure water (Mili-Q®) were also used. Potassium phosphate (Vetec®, lot 1007140) and sodium hydroxide (Sigma Aldrich®, batch SLBM7637) were used for the preparation of the dissolution medium.

### Development and obtainment of BNZ:ZIF-8 systems

The obtaining method was based on ex situ from Horcajada and collaborators [21], which promoted the adsorption of the drug using the pre-existing ZIF-8. The procedure used to produce systems in different molar ratios of BNZ: ZIF-8 (1:3, 1:1, 3:1, and 6:1) based on the molecular weights of BNZ and ZIF-8, which are 260.25 and 229.61 g.mol^−1^, respectively.

Initially, the drug was solubilized in acetone in erlenmeyers with a capacity of 250 mL, yielding an initial concentration of 20 μg.mL^−1^. The BNZ solution was sonicated for 10 min in a Limp Sonic® sonicator to ensure complete solubilization. At the end of the shaking, the ZIF-8 was suspended in acetone and added to the BNZ solution. Subsequently, the volume was filled with the respective solvent, subjecting the mixture to intermittent stirring on Magnetic Stirrer MA089 Marconi® with the aid of a magnetic bar for up to 7 days.

The supernatant was collected daily (0.33 mL, followed by solvent replenishment) and filtered through a hydrophobic filter with 0.22 μm pore aperture for further quantification by absorption spectroscopy in the Ultraviolet–Visible region (UV–Vis) with the aid of a previously performed calibration curve (Line equation: *Y* = 0.0316. *X* = 0.0003; *R*^2^ = 0.9994) for the elaboration of a BNZ incorporation curve into the ZIF-8 network. This process was used to determine the maximum incorporation efficiency (IE%) (Eq. 1).

1 $${\mathrm{IE\% = }}\frac{{\left[ {{\mathrm{BNZ}}\,{\mathrm{theoretical}}\,{\mathrm{concentration}}} \right]{\mathrm{.}}\left[ {{\mathrm{BNZ}}\,{\mathrm{real}}\,{\mathrm{concentration}}} \right]}}{{\left[ {{\mathrm{BNZ}}\,{\mathrm{theoretical}}\,{\mathrm{concentration}}} \right]}} \times {\mathrm{100}}$$

At the end of the process, the material was centrifuged at 2000 rpm for 20 min to remove residual BNZ. The supernatant was discarded and the precipitate was washed with acetone. The same procedure was repeated twice. Then, the material was dried in a drying oven with 35 °C airflow (Shel Lab®) until it was completely dry (about 4 h for oven drying). Once dried, the material was used to quantify the IE%.

This entire procedure was done in triplicate and was performed away from the light due to the inherent photosensitivity of the BNZ molecule as observed in previous studies [22]. Due to the presence of the acetone, the laboratory glassware was sealed in order to reduce the volatilization of the solvent. However, the volatilized content could be constantly restored after verification of mass loss, based on the value of 0.79 g.mL^−1^ for the density of the acetone.

For comparison purposes, Physical Mixtures (MF) in the 1:1 molar ratio (mol/mol) were obtained by simple agitation (5 min) in penicillin flasks. The results obtained from MF are shown in the Supplementary information.

### Systems characterization

The materials were characterized by several analytical techniques to confirm the formation of the BNZ:ZIF-8 systems: Scanning Electron Microscopy (SEM), X-ray Diffraction (XRD), Polarized Light Microscopy (MLP), Thermal Analysis: Thermogravimetry (TG), Calorimetry Differential Scanning (DSC), Fourier transform infrared absorption spectroscopy (FTIR), Laser particle size and surface area analysis, and pore size and volume. Some of the techniques for the work are described in the Supplementary information. However, XRD and SEM are considered gold standard techniques for the purpose of confirming the formation of DDS and, therefore, are described below.

XRD analysis was performed on a SHIMADZU® XRD-7000 diffractometer, equipped with a copper anode at a scanning speed of 1.2 °.min^−1^, in the 2θ angle range from 5° to 45°. Basal spacing was calculated using the Bragg’s equation.

To perform SEM analysis, the samples were prepared on double carbon tape contained in a copper stub and metallized under vacuum with the deposition of a thin layer of gold on BAL-TEC® equipment, model SCD 050. SEM analyzes were obtained on a SHIMADZU® equipment, model SS-550, with tungsten filament and coupling for energy dispersion (EDS), using a 300 and 750-fold increase.

### In vitro dissolution test under sink conditions

The in vitro dissolution tests were carried out at different pHs in order to evaluate the release profile of the BNZ from the obtained DDS. The assays were run at 37 ± 0.5 °C using 500 mL of phosphate buffers (pH 4.5 and 7.6) as dissolution medium, apparatus 2 (paddle) and rotational speed 75 rpm, using a Varian dissolver ® VK 7010. The dissolution medium was chosen based on recent studies showing the modulation of pH-sensitive drug release from the ZIF-8 network [23–25]. A more acidic pH was not used due to the rapid structural dissociation of ZIF-8, which would result in a rapid release of the drug [26].

Amounts equivalent to 36.66 mg of BNZ were weighed in the Shimadzu® scale of the AUX 220 where, if completely dissolved in a volume of 500 mL, it would have a concentration equal to 73 μg.mL^−1^, thus establishing the sink condition. Both sink and non-sink conditions were determined based on a quantitative solubility assay of BNZ performed by our research group where the maximum solubility in the dissolution medium used (about 240 μg.mL^−1^) was measured after 7 days of intermittent stirring (results not shown). Dissolution experiments were performed under the suitable conditions described by Rohrs [27] and sink index, which employs release medium volumes of threefold to tenfold of the volume present in the saturated BNZ solution for sink condition, and a smaller medium volume for non-sink condition.

The data collection was made at predefined time intervals of 0.08, 0.25, 0.5, 0.76, 1, 2, 3, 4, 5, 6, or 7 h or until there was a drug release of up to 80%. Five milliliters of the samples were collected, filtered through a 0.20 μm membrane filter, and subsequently diluted with dissolution medium for quantification of BNZ content by UV–Vis mini Spectroscopy at 323 nm (Model 1240, Shimadzu®) using a previously validated analytical methodology [28]. The dissolution medium was replenished with the same volume after each aliquot removed. All assays were performed in triplicate.

Regarding data analysis, these were corrected for volume and for drug loss during collection through Eq. (2) [29, 30].

2 $${\it{Ccor}} = {\it{Cn}}\frac{{{\it{Vs}}}}{{{\it{Vt}}}}\mathop {\sum}\limits_1^{n - 1} {{\it{Ci}}}$$

where *C*_n_ is the concentration at collection point *n*, *V*_t_ is the initial total volume, *V*_S_ is the volume of the collections, and *C*_i_ is the concentration of the samples at the points prior to *n*.

The dissolution profiles were evaluated and compared using the area parameter under the curve (AUC) and the percentage of drug dissolved per collection time. The AUC were obtained using Origin® 8 software from Origin Lab Solutions and Microsoft Excel® 2007, and these results were compared based on the statistical analysis performed using one-way ANOVA, in addition to standard deviation (SD).

Aiming to analyze whether there was modulation of drug release, a comparison was made between the release profiles through the model-dependent and model-independent methods. For the model-dependent method, the adjustment was analyzed for the following models: zero order, first order, Higuchi, Korsmeyer–Peppas, and Peppas–Sahlin. The regression equation of the line was determined through trend lines of the corresponding graphs following the general formulas presented in Supplementary Table 1.

The most relevant mathematical model was chosen from the coefficient of determination (*R*^2^) and the adjusted coefficient of determination (*R*^2^_adjusted_). These have different parameter numbers, which can increase the value of *R*^2^ due to over-adjustments. In this context, the adjusted *R*^2^ tends to reduce this over-adjustment, and is therefore the coefficient used to choose the best model. *R*^2^_adjusted_ is calculated (Eq. 3) and the closer the numerical value of 1, the better the sample apllies to a determinated model. Calculations were performed using Microsoft Excel® 2007 software and the DDSolver®add-in [30, 31]

3 $$R_{\mathrm{adjusted}}^2 = 1-\frac{n-1}{n-p}x\left( {1-r^2} \right)$$

where *n* is the number of points in the sample, *p* is the number of model parameters, and *r*^2^ is the coefficient of determination.

For the model-independent method, the similarity factor (ƒ2), was used. The ƒ2 is a reciprocal logarithmic transformation of the square root of the sum of the quadratic error and is a measure of similarity of the percentage of dissolution between two curves (Eq. 4) [32, 33]:

4 $$f2 = 50.log\left\{ {\left. {\sqrt {\left[ {1 + \left( {\frac{1}{n}} \right)\mathop {\sum}\limits_{t = 1}^n {\left( {Rt - Tt} \right)^2} } \right]^{ - 0,5}} \times100} \right\}} \right.$$

where *n* is the number of collection times considered for calculation purposes; Rt is the percentage value dissolved at time *t* obtained with the reference or comparator curve; and Tt is the percent dissolved value of the test curve, or the altered formulation at time *t*.

### In vitro dissolution test in non-sink conditions

In vitro dissolution tests in non-sink conditions were performed using the same equipment and data analysis described in topic 2.4. However, for the maintenance of the non-sink condition, smaller amounts of drug and medium were used.

In the study, samples of 27.5 mg of BNZ were used where, if completely dissolved in a 250 mL volume of dissolution medium, the concentration would have been 110 μg.mL^−1^ (*sink* index <3), thus establishing the non-sink condition, also based on the quantitative solubility assay of BNZ. For MF and BNZ:ZIF-8 systems, samples with a 1:1 molar ratio were used.

## Results

### Evaluation of drug incorporation and characterization of BNZ@ZIF-8 DDS

Based on the UV–Vis spectra (200–1000 nm) (Supplementary Fig. 1), the BNZ solution (20 μg.mL^−1^) had maximum absorption at 323 nm, and the ZIF-8 at 243 nm. And at 323 nm, the ZIF-8 presents a value of 0.004 of absorbance.

Thus, it was possible to construct daily calibration curves based on the method developed by Silva and collaborators [28], making possible the calculation of the apparent incorporation of the BNZ into the ZIF-8 network through Eq. (1).

**Fig. 1:**
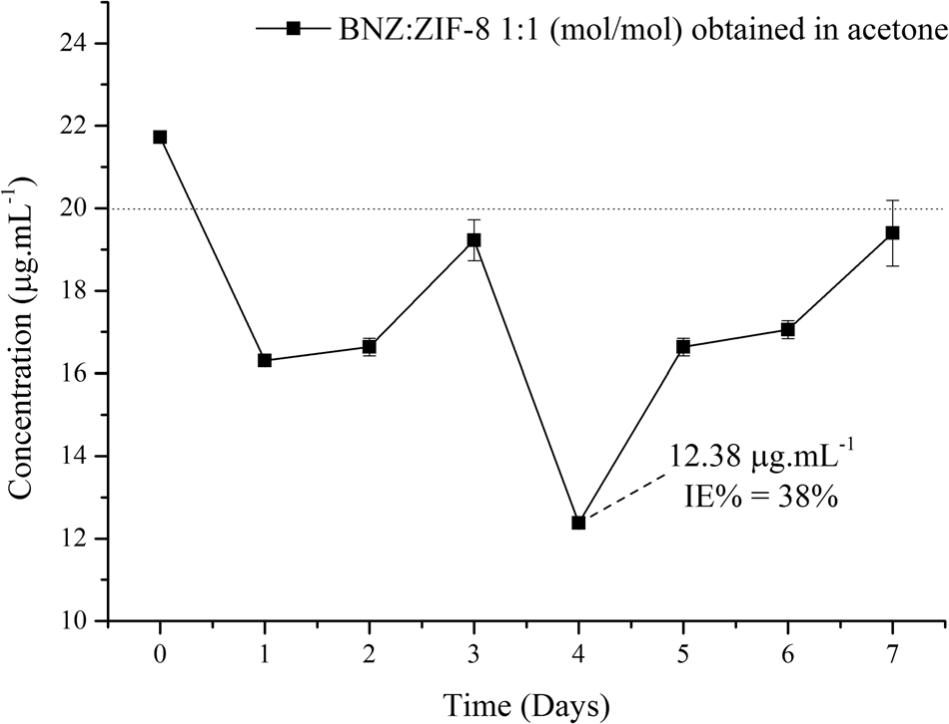
BNZ incorporation curves into the ZIF-8 network obtained in acetone in a 1:1 molar ratio (the dashed line refers to the initial concentration; *IE%* incorporation efficiency)

From the daily calibration curves, it was calculated the apparently incorporation of the BNZ into the ZIF-8 network. The experiment aimed at the decay of the drug concentration, justified by its incorporation into the ZIF-8 mesh. Regarding the incorporation curves (Fig. 1), it was possible to observe that, after four days of stirring, the system presented a lower concentration point, totaling approximately 38% of the incorporation. The theoretical initial concentration was 20 μg.mL^−1^, and the actual concentration was 12.83 ± 0.98 μg.mL^−1^.

Thus, in order to corroborate the obtained results, the sample was redispersed in the respective solvent (acetone) to remove the residual BNZ, followed by centrifugation at 2000 rpm for 20 min. After centrifugation, the supernatant was discarded and the precipitate was resuspended again to determine the BNZ concentration, which in turn showed a IE% = 34%. The experiments, which were performed in triplicate, demonstrated low standard deviations of the isolated points, evidencing the reliability and reprodutibility of the results.

Thus, new DDS were obtained at the end of the fourth day of intermittent stirring, followed by pertinent characterizations. In order to corroborate the reprodutibility of the results, systems were obtained only with the same physicochemical properties, thus guaranteeing the continuous production of the materials.

In order to optimize the obtaining method, different molar ratios (1:3, 3:1, and 6:1 mol/mol) were evaluated in order to identify the one with the highest IE%. However, the values were all below that of the molar ratio of 1:1 (0.0, 21.07, and 23.57, respectively), as observed in Supplementary Fig. 2.

In this way, the BNZ:ZIF-8 (1:1 molar ratio) system was selected to continue the work, since it presented a higher IE% than the other proportions. This was then named BNZ@ZIF-8 and it was the objective of a several characterization techniques, which suggest the BNZ physical adsorption to the coordination network of ZIF-8. The characterization techniques are detailed in the Supplementary information, but, to confirm the obtainment of the new DDS entity, XRD and SEM have been elucidated.

#### X-ray diffraction

It was possible to identify the characteristic peaks of both BNZ crystals (7.36°, 16.28°, and 21.86°) and ZIF-8 (7.44°, 10.46°, and 12.78°, corresponding to reflections 011, 002, and 112, relating to a body-centered hub). These data were used to calculate the basal spacing in order to establish a reference value to characterize the insertion of the BNZ molecule in ZIF-8. These results are in line with recent works [14, 22].

The BNZ@ZIF-8 system also presented crystalline behavior, resulting in the sum of the XRD profiles of the isolated substances. However, different from MF, the system showed the most characteristic peaks of the BNZ (7.36°, 10.88°, 16.82°, and 21.88°), which suggests the formation of the coupled system, even though it is only characterized by the physical adsorption of the BNZ on the surface of ZIF-8.

**Fig. 2:**
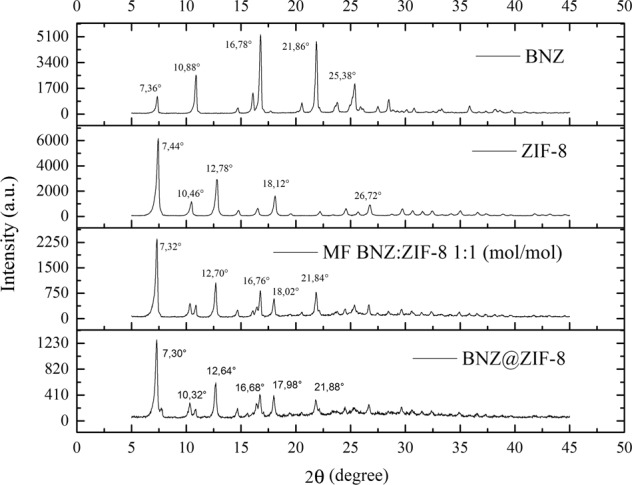
XRD of the BNZ, ZIF-8, MF, and the BNZ@ZIF-8 with their respective main peaks

In this material, a reduction of the peak intensity characteristic of ZIF-8 (7.44°) was observed indicating the strong attraction between the BNZ and the ZIF-8 network. Similar results were evidenced by Liédana and collaborators [23]. It is important to note that the obtained XRDs demonstrate that the structural integrity of ZIF-8 remains unchanged after the adsorption of the BNZ. This is of paramount importance because it shows that even though the formation of the systems promotes some reduction of the peaks of the molecule, the crystalline structural integrity of the ZIF-8 is maintained [15, 16]. The XRD results of all materials are summarized in Fig. 2.

Basal spacing values, defined by the Bragg’s equation, were used as a parameter to confirm the insertion of the drug into the ZIF-8 molecule. Values greater than baseline suggest that the drug was inserted into the network and, therefore, dilates the space between the lamellae. However, the discrete increase in basal spacing was observed when the isolated ZIF-8 was compared to the MF and to the BNZ@ZIF-8 (Table 1), mainly at the peak of reflection 110, the most characteristic of ZIF-8 crystallinity and of the rhombic dodecahedral of its crystals [25]. This fact corroborates the system’s formation but suggests that it is the product of drug adsorption to the surface of the ZIF-8, characterizing a physical interaction, since the increase of the basal spacing is very small.

**Table 1:** Calculation of the basal spacing of the reflection peaks 110, 002, and 112 of ZIF-8, Physical Mixture, and BNZ@ZIF-8 obtained in water and acetone

Sample	Peak (°2θ)	Reflection	Basal spacing (Å)
ZIF-8	7.44	110	11.87
	10.46	002	8.71
	12.78	112	6.94
MF	7.32	110	12.04
	10.4	002	8.51
	12.70	112	6.97
BNZ@ZIF-8	7.3	110	12.11
	10.32	002	8.10
	12.7	112	6.97

#### Scanning Electron Microscopy (SEM)

**Fig. 3:**
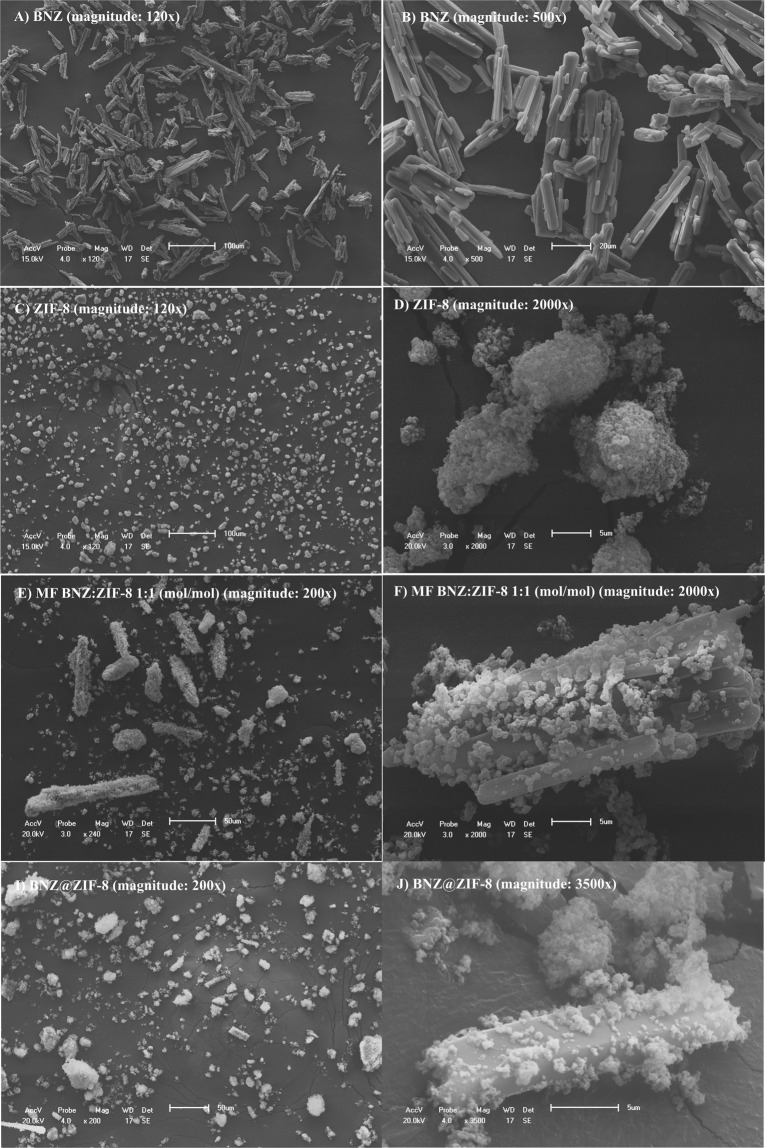
Scanning Electron Microscopy of (**a**) and (**b**) BNZ; (**c**) and (**d**) ZIF-8; (**e**) and (**f**) physical mixtures; (**g**) and (**h**) and BNZ@ZIF-8 system

The SEM of the drug demonstrated the regular crystalline appearance of the BNZ (Fig. 3a, b) corroborating with the XRD. Most crystals had irregular sizes of approximately 100 μm. It was observed that the ZIF-8, also crystalline, presents very small size and dodecahedral (almost spherical) aspect (Fig. 3c, d) and appear accompanied by clusters of nanocrystals [13]. The MF and BNZ@ZIF-8 images confirmed the information suggested by the XRD about the physical adsorption between the drug and the ZIF-8 surface. It is possible to observe the deposition of the ZIF-8 spherical crystals on the surface of the regular BNZ crystals (Fig. 3e, f).

On the other hand, the BNZ@ZIF-8 system showed a reduction in size and change in the morphology of the regular BNZ crystals Fig. 3g, h). Although still demonstrating the crystallinity and physical interaction of adsorption between the drug and ZIF-8, the reduction of crystal size corroborates the results of XRD regarding the formation of DDS. This may indicate an enhancing of aqueous solubility of the BNZ due to the increase in surface area and consequent higher degree of solvation of the drug crystal. This increase in aqueous solubility and dissolution rate can be confirmed by elucidating the DSC curve—reduction of Ea required for drug fusion—and further in vitro dissolution test [27].

### In vitro release assay

#### Dissolution test under sink condition

**Fig. 4:**
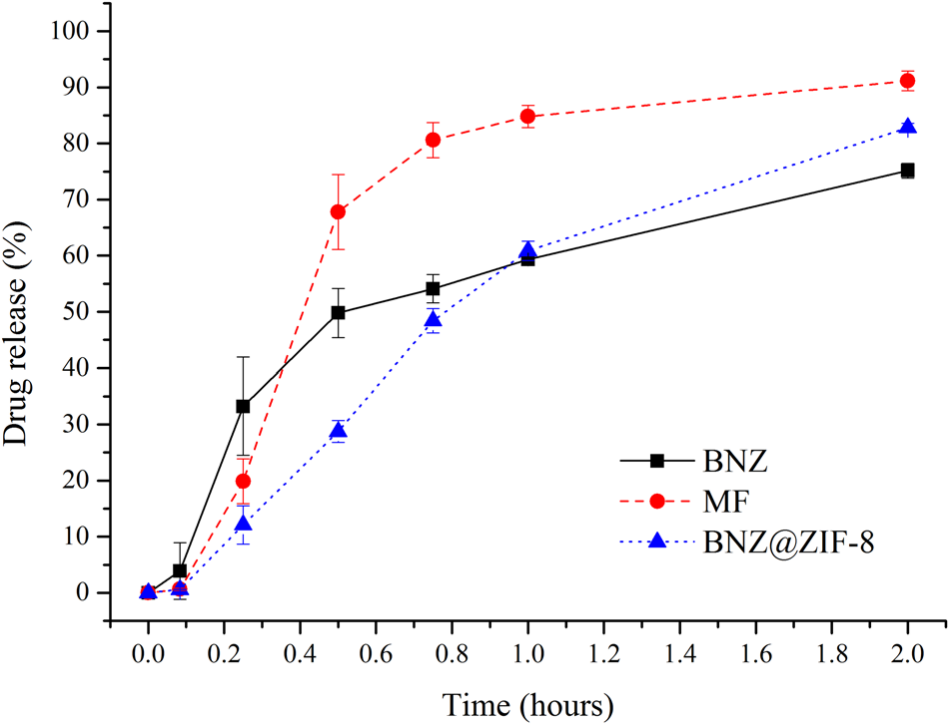
Dissolution profile in sink conditions of BNZ, MF, and BNZ@ZIF-8 system at a pH of 4.5

At a pH of 4.5 (Fig. 4), the isolated BNZ completes the experiment with a lower percentage of drug released when compared to the BNZ@ZIF-8, a good part of which has already dissolved at the beginning of the dissolution, a phenomenon known as burst effect. MF is able to achieve an 80% release rate in 45 min, adapting to a conventional release. BNZ@ZIF-8, on the other hand, showed a better modulation of drug release. In 30 min, for example, the BNZ and MF already have released approximately 50 and 68% of the drug, respectively, while the BNZ@ZIF-8 has only released 27%. After two hours, the release of the drug in BNZ@ZIF-8 reached 83%, thus, was also possible to observe the discrete increment of the solubility of the drug, since in two hours the percentage of isolated BNZ dissolved was only 75%.

**Fig. 5:**
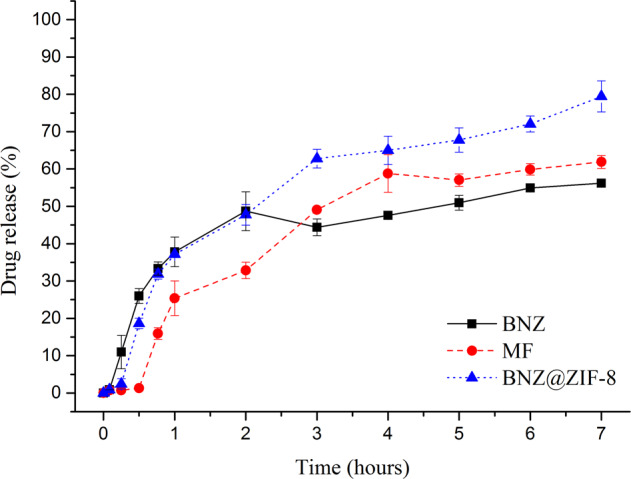
Dissolution profile in sink condition of the BNZ, MF, and BNZ@ZIF-8 system at a pH of 7.6

In parallel, it was verified that under a pH of 7.6 (Fig. 5), BNZ@ZIF-8 obtained percentages of drug release superior to both isolated BNZ and MF. In the first 30 min of dissolution, it is possible to identify that the isolated BNZ begins to obtain a high rate of release, while the MF and BNZ@ZIF-8 retain the drug. From then on, a rapid liberation of the BNZ occurs. In these results, it was also seen that MF behaved with a gradual release as time increased. However, the BNZ@ZIF-8 showed a more balanced modulation of the drug release, where in 7 h it reached the maximum point of about 80% of the drug, higher than the MF (61%) and isolated BNZ (56%).

The results contained in Table 2 showed that the AUC values of the majority of MF time intervals at a 4.5 pH were higher when compared to the isolated BNZ. In addition, comparing the AUCs of MF and BNZ with the BNZ@ZIF-8, maintaining the same molar ratio of BNZ, it is observed that the values in BNZ@ZIF-8 are always lower, which corroborates the dissolution profile results regarding the modulation of drug release. From the second collect on, comparing MF with BNZ @ ZIF-8, the data obtained were statistically different when they were treated by one-way ANOVA (*p* = 0.05*).

**Table 2:** Mean values of AUC of BNZ, MF, and BNZ@ZIF-8 system in sink conditions and a pH of 4.5

AUC (min.µL).mL^−1^ em pH 4.5			
Time (hours)	BNZ	MF	BNZ@ZIF-8
0.083	0.16	0.02	0.02
0.25	2.29 ± 0.55	1.44 ± 0.72*	0.87 ± 0.29*
0.5	11.23 ± 1.57	11.96 ± 3.39*	5.51 ± 0.96*
0.75	24.22 ± 2.43	30.51 ± 5.32*	14.29 ± 2.18*
1	38.39 ± 2.71	51.19 ± 5.96*	27.10 ± 3.37*
2	105.63 ± 1.90	139.18 ± 7.22*	98.62 ± 3.83*

*p ≤ 0.1

In parallel, at a pH of 7.6 (Table 3), until the first 3 h, BNZ@ZIF-8 obtained AUC values lower than the isolated BNZ, showing the modulation of the drug release that occurs in a prolonged way. After this time, values significantly higher than those presented by the isolated drug were observed, showing the success of BNZ@ZIF-8 in not only modulating the drug release, but also in enhancing BNZ aqueos solubility. The values obtained from 1 h of drug release onwards were statistically different (when treated by one-way ANOVA with *p* = 0.05**).

**Table 3:** Mean AUC values of BNZ, MF, and BNZ@ZIF-8 system at a pH of 7.6 under sink conditions

AUC (min.µL).mL^−1^ em pH 7,6			
Time (hours)	BNZ	MF	BNZ@ZIF-8
**0.083**	0.03	0.03	0.03
**0.25**	1.02 ± 0.38	0.15 ± 0.19	0.31 ± 1.12
**0.5**	5.64 ± 1.19	0.41 ± 0.02	3.04 ± 2.04
**0.75**	13.46 ± 1.81	2.71 ± 1.86	9.77 ± 6.75
**1**	21.68 ± 2.59	7.52 ± 4.92**	17.12 ± 11.20**
**2**	64.93 ± 7.18	32.96 ± 17.58**	56.90 ± 28.35**
**3**	110.80 ± 11.76	70.25 ± 23.68**	110.50 ± 44.15**
**4**	156.12 ± 14.09	124.17 ± 20.91**	172.73 ± 57.86**
**5**	205.41 ± 14.45	182.08 ± 17.56**	239.10 ± 70.87**
**6**	258.36 ± 16.68	240.53 ± 15.98**	305.84 ± 79.55**
**7**	313.90 ± 17.30	301.42 ± 14.35**	378.40 ± 86.57**

**p ≤ 0.05

In order to ensure that there was a modulation of BNZ release at different pH levels, a study of the drug release kinetics was carried out through different model-dependent and model-independent methods.

Observing the values of *R*^2^_adjusted_, the modulation of the drug release could be evidenced by comparing the isolated BNZ and the BNZ@ZIF-8, since the materials showed adjustments to different kinetic models (Supplementary Table 3). As the BNZ@ZIF-8 system presented more interesting results, the MF was not taken in consideration in these tests. At pH 4.5, BNZ demonstrated applicability to the Korsmeyer–Peppas method and BNZ@ZIF-8 to the first-order model. At pH 7.6, BNZ continued to adjust to the same model, while BNZ@ZIF-8 adapted to the Peppas–Sahlin model, proving the modulation of the release.

Thus, comparing the different pH to verify the drug-dependent release from the BNZ@ZIF-8, at pH 4.5, the more rational kinetic models for BNZ@ZIF-8 were, in descending order: First Order > Peppas–Sahlin > Korsmeyer–Peppas > Zero Order > Higuchi. At pH 7.6, the system obeyed kinetic models in the following order: Peppas–Sahlin > First Order > Korsmeyer–Peppas > Higuchi > Zero Order.

Then, the release coefficient (*n*) was calculated through the Korsmeyer equation. In the dissolution conditions studied, one of them (obtained from the linear regression equation) was found to have value above 1 (Supplementary Table 4), being classified as super case II transport, where the diffusion rate is superior to that of relaxation, being controlled by the velocity of migration of the border between the carrier and the nucleus, corroborating with the results found by the Peppas–Sahlin model [34, 35].

Another method applied to compare the similarity between the dissolution profiles was the simple independent model by calculating the similarity factor. The ƒ2 is indicative of the similarity between the dissolved percentages of both profiles, confirmed when the value is between 50 and 100 [33]. As shown in Supplementary Table 5, the values resulting from the comparison of the dissolution profiles between BNZ and MF and between BNZ and BNZ@ZIF-8, both at pH 4.5 and 7.6, were lesser than the range between 50 and 100, revealing that there was, in fact, a modulation of drug release.

#### Dissolution test under non-sink condition

**Fig. 6:**
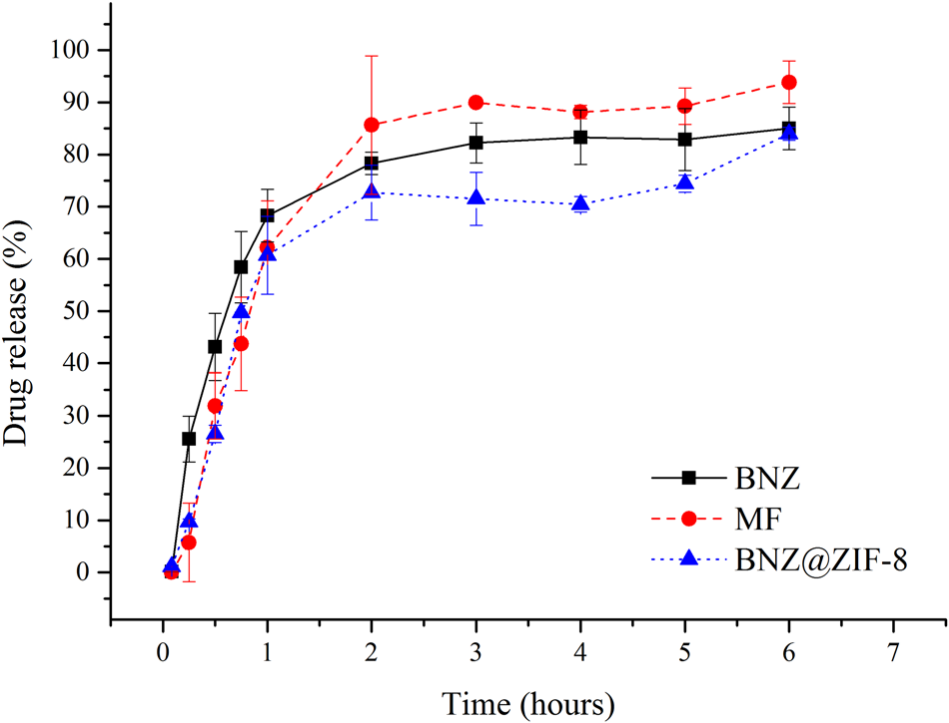
Dissolution profile in non-sink conditions of BNZ, MF, and BNZ@ZIF-8 system at a pH of 4.5

Under the non-sink condition, at pH 4.5 (Fig. 6), isolated BNZ shows rapid burst release in the first hour of dissolution, reaching approximately 64% of drug released. Thereafter, the drug is released gradually, reaching 80% in three hours. This concentration remains practically in a plateau until the end of the experiment, suggesting the reach of the saturation concentration of the dissolution medium.

In parallel, MF and the BNZ@ZIF-8 present similar profiles, however, these demonstrate the reduction of the burst effect, releasing the drug more slowly. In addition, in the first hour of dissolution, the percentage released is practically the same for the isolated BNZ, MF, and BNZ@ZIF-8. From there on the materials have different behaviors. MF releases the drug rapidly, reaching 96% of drug released at the end of the experiment, which corresponds to a solubility 8.31% higher when compared to the isolated BNZ. This provides MF a discrete enhance of solubility, probably, by stabilizing the dissolution of the drug.

BNZ@ZIF-8 reaches 80% of drug released only six hours of dissolution, more slowly, characterizing a prolonged release. It is worth noting that the experiment ends with an upward curve, suggesting that a higher percentage of drug released would be achieved if the experiment was followed. These results are interesting because, as seen in the dissolution studies carried out in the sink condition at more acidic pH levels, the ZIF-8 expands and/or breaks down, which could promote a much faster release of the drug.

**Fig. 7:**
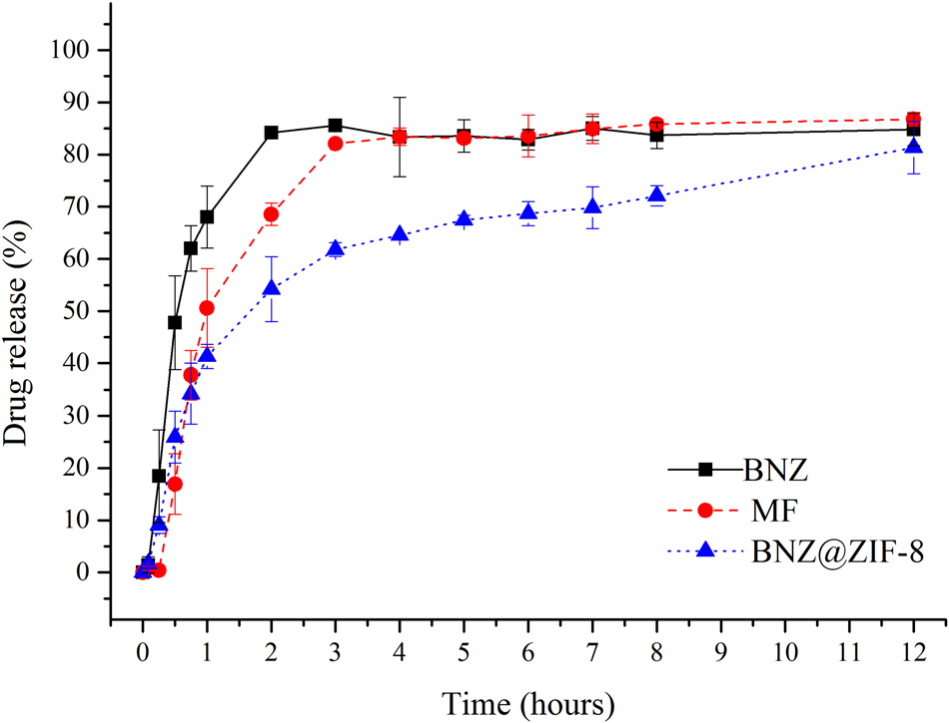
Dissolution profile in non-sink conditions of BNZ, MF, and BNZ@ZIF-8 system at a pH of 7.6

At pH 7.6 (Fig. 7), the isolated BNZ presents an intense burst effect, achieving a 63% release in the first hour of dissolution. From this point on the release occurs quite quickly, reaching 80% in the second hour before coming to a plateau that corresponds to the saturation of the dissolution medium, where it remains in equilibrium until the end of the experiment.

The MF presents a reduction of the burst effect and the release occurs more slowly than the isolated drug. In the first hour of dissolution, it presents only 47% of the drug released and, from there, it presents a slow and gradual release, showing lower values up to the three hours, where it reaches a released percentage similar to the isolated BNZ, remaining at this level also to correspond to the saturation of dissolution medium. In this case, the MF does not seem to show results of BNZ’s solubility enchancing.

BNZ@ZIF-8 system has drastically reduced the burst effect exhibited by other materials, presenting a more slow release. In the first hour of dissolution, only 38% of the drug is released (almost half of what has been observed). Prolonged drug release is observed throughout the course of dissolution, slowly but steadily, reaching 80% drug release only after 12 h of experiment. It is worth noting that the graph shows the end of the experiment still in an upward curve, suggesting the continuity of the asset release over time. These results, in fact, shows the promising pH-sensitive effect of this DDS, especially when analyzing that the *non-sink* condition might be observed in gastrointestinal tract (GIT) and that this directly affects the absorption of oral solid dosage forms.

These results are corroborated by an AUC analysis (Tables 4 and 5). For both pHs used in the dissolution tests under the *non-sink* condition, from the second collect onwards all AUC values were statistically different (with *p* = 0.05). It is possible to observe that, at pH 4.5, the AUC values of the MF and BNZ@ZIF-8 are always lower than the values observed for the isolated drug; but they are very close. However, after three hours, BNZ@ZIF-8 has lower AUC values than the isolated drug and MF, demonstrating that the material is able to modulate the release of the drug, while the MF remains with similar values to the BNZ alone. These results were already expected due to the dissociation of the ZIF-8 network structure called breathing phenomenon.

**Table 4:** Mean AUC values of BNZ, MF, and BNZ@ZIF-8 system at a pH of 4.5 under non-sink conditions

AUC (min.μL).mL^−1^ in pH 4,5 (*non-sink* conditions)			
Time (hours)	BNZ	MF	BNZ@ZIF-8
0.083	0	0	0
0.25	1.70 ± 0.84	0.44 ± 0.61	0.82 ± 1.81
0.5	9.16 ± 2.77	4.16 ± 3.23	4.96 ± 4.69
0.75	21.20 ± 4.15	12.81 ± 6.91	13.68 ± 6.36
1	36.25 ± 5.41	26.14 ± 10.91	26.97 ± 7.16
2	105.94 ± 7.96	94.43 ± 25.56	91.91 ± 15.56
3	183.18 ± 7.50	177.57 ± 33.56	160.82 ± 25.03
4	265.74 ± 5.64	263.96 ± 40.75	230.66 ± 31.10
5	347.76 ± 4.65	352.56 ± 48.31	302.24 ± 33.97
6	433.83 ± 4.63	446.21 ± 52.08	383.42 ± 39.50

**Table 5:** Mean AUC values of BNZ, MF, and BNZ@ZIF-8 system at a pH of 7.6 under non-sink conditions

AUC (min.μL).mL^−1^ in pH 7,6 (*non-sink* conditions)			
Time (hours)	BNZ	MF	BNZ@ZIF-8
0.08333	0	0	0
0.25	1.25 ± 1.10	0.09 ± 0.03	0.82 ± 0.76
0.5	8.04 ± 3.99	1.50 ± 1.64	4.81 ± 2.47
0.75	20.25 ± 5.58	7.16 ± 3.84	11.70 ± 3.67
1	35.27 ± 5.64	17.30 ± 3.49	20.44 ± 5.30
2	106.96 ± 16.55	72.30 ± 0.78	64.87 ± 13.53
3	187.47 ± 36.70	142.27 ± 3.49	119.15 ± 26.00
4	267.83 ± 40.67	219.82 ± 5.95	178.76 ± 38.62
5	348.25 ± 45.94	299.94 ± 8.07	241.68 ± 48.05
6	452.17 ± 43.14	404.47 ± 3.29	307.23 ± 58.26
7	536.10 ± 43.83	488.72 ± 6.72	374.64 ± 69.22
8	620.43 ± 43.81	574.09 ± 8.58	444.40 ± 77.23
12	957.56 ± 45.4	919.20 ± 7.76	747.77 ± 94.31

At 7.6 pH the MF has lower AUC values on regards both to the isolated BNZ and BNZ@ZIF-8 up to the one-hour mark. Thereafter, the MF resembles the isolated drug in the dissolution medium saturation condition. From the 2-h mark, BNZ@ZIF-8 presents significantly smaller values until the end of the experiment. In addition, even as BNZ and MF reach dissolution medium saturation, BNZ@ZIF-8 continues to release the drug for up to 12 h (where it reaches 80%). Thus, it is evident that, in addition to prolonging the release of BNZ, the BNZ@ZIF-8 could enhance its aqueous solubility and, consequently, its bioavailability, stabilizing its dissolution and increasing its dissolved content in non-sink conditions such as those observed during the absorption drugs from oral solid dosage forms.

The same release kinetic models applied for sink conditions were also used to corroborate the obtainment of modulated release profiles in non-sink conditions. The *R*^2^_adjusted_ was also adopted in order to reduce the over-adjustment of the methods. As verified, the models that applied to the dissolutions at pH 4,5 and 7,6 were Korsmeyer–Peppas and Peppas–Sahlin, respectively.

The *R*^2^_adjusted_ values showed the modulation of the drug release comparing the isolated BNZ and the BNZ@ZIF-8, since they present adjustments to different models (Supplementary Table 6). At pH 4.5, BNZ demonstrated applicability to the Peppas–Sahlin method, and BNZ@ZIF-8 to Korsmeyer–Peppas. At pH 7.6, the isolated dissolution of BNZ adjusted to another model, the Korsmeyer–Peppas, showing a different behavior from that observed in the sink condition, while the BNZ@ZIF-8 dissolution adapted to the Peppas–Sahlin model.

Then, the pH-sensitive drug release from the BNZ@ZIF-8 was analyzed by comparing the kinetic models between the different pHs values. For the dissolution in pH 4.5, the most applicable kinetic models for BNZ@ZIF-8 were, in descending order: Korsmeyer–Peppas > Peppas–Sahlin > Higuchi > First order > Zero order. At pH 7.6, BNZ@ZIF-8 obeyed the kinetic models in the following decreasing order: Peppas–Sahlin > First Order > Korsmeyer–Peppas > Higuchi > Order zero.

Regarding the model-independent method, the MF and BNZ@ZIF-8 release profiles at pH 4.5 were similar to the isolated BNZ release profile, since the values of ƒ2 were between 50 and 100 for both materials. However, at pH 7.6, there was a modulation of the release, since the release profiles of the materials are different from the profile presented by the isolated BNZ (ƒ2 values <50). This shows that, at acidic pH, the BNZ@ZIF-8 behaves similarly to isolated BNZ, releasing the drug rapidly and at pH 7.6 it promotes a more prolonged and pH-sensitive release.

## Discussion

Although in the UV–Vis scan it was observed that the ZIF-8 also exhibits an absorption peak in the UV–Vis region, the method did not present selectivity problems because the material does not absorb at the same wavelength of the BNZ. In addition, ZIF-8 is insoluble in water and apolar solvents, excluding it from the analyte at the time the sample is filtered, as evidenced by Alves [36].

From the incorporation curves (Fig. 1), was observed that the system obtained presented a lower concentration point after 4 days of stirring, totaling ~38% of the incorporation, a higher percentage of incorporation when compared with results were evidenced by Zhuang and collaborators [18]. However, this value may comprise not only the drug complexed to the ZIF-8 pores, but also that physical absorved to the surface thereof. But it was possible to prove that even after centrifugation the incorporation efficiency was 34%, suggesting that there is a strong bond between the drug and ZIF-8. Similar values were evidenced by Liédana and collaborators [23]. In this way, the system obtained in acetone in the 1:1 molar ratio was selected to proceed to the characterizations and dissolutions studies, since it presented a higher IE% than the other proportions.

Regarding the dissolution assays at sink condition, according to Zhuang and collaborators [18], this quick release in 2 h at pH 4.5 is due to the decoupling of ZIF-8 coordination networks at an acidic pH, which causes the network to disintegrate and the drug to be released faster. While at pH 7.6 the BNZ@ZIF-8 system is capable of not only modulating the drug release in order to maintain it in constant concentrations—inferring the safety of the treatment in relation to a lower oscillation of the concentration within the therapeutic window—but also of enhancing the drug aqueos solubility, since the AUCs values were different.

Comparing the two drug release profiles, it can be seen that at a pH 4.5, the structure of ZIF-8 dissociates more quickly, releasing a large amount of drug in a short time. On the other hand in the dissolution medium at pH 7.6, the BNZ release through BNZ@ZIF-8 occurs in a prolonged way, increasing gradually according to the time intervals. Similar results, however, with different drugs carried were evidenced by Vasconselos and collaborators and Sun and collaborators [24, 37].

In both profiles (Figs. 4 and 5) it is possible to observe that MF also presented a pH-sensitive release profile. However, this shows that, in fact, the simple physical adsorption of BNZ to ZIF-8 is enough to promote sufficiently strong interaction to modulate drug release, although less intense than the profile evidenced by the BNZ@ZIF-8. Therefore, MF was not taken into consideration in some tests.

In order to verify the results of the dissolution profiles, AUC were calculated (Tables 2 and 3). These values represent the maintenance of supersaturation, which is achieved through the physical interactions and/or chemical bonds between the carrier and the drug molecules, thereby inhibiting drug precipitation and enhancing their levels in solution, ensuring improved aqueous solubility and, hence, its bioavailability [37, 38]. At pH 4.5, BNZ@ZIF-8 showed lower AUC values than isolated BNZ and MF showing modulation of drug release in a prolonged term. While at pH 7.6 after 3 h of dissolution it is observed that AUC of BNZ@ZIF-8 was superior to isolated BNZ and MF demonstrating that in addition to modulating the release of the drug it also increased its aquoes solubility in a discrete way.

This result is likely due to the stabilization of drug dissolution. As evidenced by the SEM and other characterizations, in the BNZ@ZIF-8, the BNZ recrystallizes to a smaller size when compared to the isolated drug. This increases the dissolution rate thereof. Although it does not show significant results of increased solubility at both pH levels tested, at a pH 7.6, the prolongation of the BNZ release is more accentuated, as in an acidic pH, the ZIF-8 loses its structure faster. Which was analyzed from a dependent and independent release kinetics study.

As a model-dependent method, different kinetic release models were tested. The first-order and Peppas–Sahlin models were then determined for BNZ@ZIF-8, as adjusted by *R*^2^. The values of *R*^2^ and *R*^2^_adjusted_ can be seen in Supplementary Table 4. In the first-order model, the solid particles dissolution rate in a liquid medium depends on the ratio of dissolved drug amount and the amount of drug remaining in the carrier, so that the amount of drug released decreases over time. This is a model that can occur in extended releases, but it is mainly used for conventional releases, which is confirmed by the rapid disintegration of the organometallic network at a pH of 4.5, releasing much of the BNZ in a short test time.

Regarding the Peppas–Sahlin model, the constants K_1_ and K_2_ (Supplementary Table 5) represent, respectively, the influence of carrier diffusion and relaxation on drug release. The highest value found was K_1_, meaning that the influence of diffusion on drug release is greater than the relaxation of the organometallic network itself. Thus, BNZ release occurs by diffusion, where the drug is transported from one location to another within the carrier, resulting in a set of random molecular motions that occur at small distances [34].

The release coefficient (n) was calculated through the Korsmeyer equation. This is generally used to interpret and describe drug release when the prevailing mechanism is not well known or when it results from the combination of two apparently independent processes: one due to drug transport that obeys Fick’s law or Fickian transport, and another a consequence of the swelling and relaxation phenomena [34, 35].

By observing the results obtained, it can be seen that the drug is rapidly released in an acidified environment (due to the decoupling of the coordination network). In contrast, at a higher pH (pH 7.6) a slower and more gradual release can be observed where the drug is released by diffusion. There may still be relaxation of the organometallic network, characterized by its breathing phenomenon. Consequently, incorporation of BNZ into ZIF-8 may enhance the drug aqueos solubility and therefore increase its dissolution rate. This could also increase its bioavailability and reduce the incidence of systemic side effects, since a lower dose of the drug would be necessary to reach the therapeutic window. In addition, the pH-sensitive characteristic of DDS may be the objective of studies aimed at targeting BNZ to cells infected by the parasite in Chagas disease.

The in vitro*–*in vivo correlation under the sink condition can be considered a problem, since the supersaturation capacity of the studied analytes, which can also happen in vivo, may not be visualized under these conditions. Thus, recent works have used the non-sink condition to visualize stages of supersaturation, in addition to nucleation and crystallization of drugs; these processes occur commonly in the GIT [37, 38].

Although the BNZ belongs to the BCS class II, it presented a good release profile up to 80% at pH 7.6 and thereafter, as expected from the non-sink condition, reached a plateau due to dissolution medium saturation. It is suggested that this high release rate, even in a non-sink condition, occur due to the low sink index used (2.2-fold of the volume present in the saturated BNZ solution), very close to the sink condition, being a lower value in the in vivo conditions. However, the aim of the present study was to compare the drug release profile under different dissolution conditions, and further in vivo studies are required to complement these analyzes.

Thus, it is evident that there was a pH-sensitive drug release as well as was evidenced for dissolution in the sink condition. At a pH 4.5, the release occurs rapidly due to the dissociation of the ZIF-8 network and diffusion of the drug. However, it occurs more slowly than in a sink condition, since the drug reaches 80% release in only 6 h, while at the same time BNZ@ZIF-8 has released less than 70%. At pH 7.6, the BNZ release of 80% occurs only after 12 h. This implies that the efficiency of DDS in modulating drug release as the pH changes is maintained even under the non-sink conditions, most commonly found in GIT for BCS class II drug.

As in the sink condition, MF also presented pH-sensitive release, but more discrete than BNZ@ZIF-8. In addition, the MF release profile presented a plateau related to the dissolution medium saturation, which was not evidenced by the system produced, which makes the BNZ@ZIF-8 the only material of interest for the objective proposed by the present work regarding on prolonged release.

While there was a change in fit to the kinetic model at the pH 4.5 when compared to sink conditions, at the pH 7.6 the above order was exactly the same. It is, therefore, notorious that under non-sink conditions, both analyzed pHs demonstrate kinetic models that are suitable for a release characterized as prolonged.

Korsmeyer and collaborators [35] developed the method capable of verifying the release of a drug from a polymer matrix. In the method, to determine the release mechanism it should take into consideration the first 60% of the drug release. For the case of cylindrical tablets, the value of *n* ≥ 0.45 corresponds to a Fickian diffusion mechanism, between 0.45 < *n* < 0.89, a non-Fickian diffusion, at *n* = 0.89 the polymer relaxes, and for *n* > 0.89 a super case II transport [30].

The Korsmeyer–Peppas method demonstrated good applicability since ZIF-8 is a coordination polymer matrix. In addition, the value of *n* = 1.64 corresponds to a super case II transport, suggesting that more than one mechanism may be involved in BNZ release kinetics, referring to the combination of relaxation and erosion followed by diffusion of the drug from the ZIF-8 network, with diffusion being the most significant mechanism [31, 39–41].

It is worth mentioning that, when analyzing the Peppas–Sahlin model, which also showed good applicability, the values of *K*^1^ were lower than *K*^0^, which indicates that the drug release was mainly controlled by Fickian diffusion, together with the contribution of relaxation of the polymer chain, characteristic of transport mechanism case II. A recent study by Khalid and collaborators [41] showed similar behavior when evaluating the mechanism of release of flurbiprofen-based mucoadhesive tablets.

These results found in the non-sink condition are more expressive than those obtained in sink conditions, since at an acidic pH the ZIF-8 dissociates more rapidly. Meanwhile, the dissolution medium enters the matrix, dissolves the drug, and diffuses it outwardly. Thus, it is evident that super case II transport is the most pertinent, since it is characteristic of materials that present all types of mechanism of release: swelling, relaxation, and diffusion.

At a pH 7.6, the release mechanism in non-sink conditions was the same as in sink conditions. The most appliable method was Peppas–Sahlin, with a value of K^1^ > K^2^, characterizing a more evident role of diffusion as mechanism of drug release. Moreover, when *n* is calculated through the Korsmeyer equation (Supplementary Table 7), it can be seen that the release mechanism also follows the super case II transport type (*n* > 0.89), similar to what occurred at the same pH in the sink condition and at pH 4.5 in non-sink conditions. This shows that an association of all release mechanisms is involved in the BNZ in vitro dissolution test under non-sink conditions. It is therefore noticeable that both models have been able to lengthen the drug release, since the Korsmeyer–Peppas and Peppas–Sahlin models are characteristic of prolonged-term dosage forms.

Once information about the mechanism of BNZ release from solid dosage forms has been defined, further studies can be planned. In the reproduction cycle, the parasite establishes mechanisms of action capable of favoring its entry into the host-cell cytosol which the proliferative phase occurs due to the rupture of the phagocytic vacuole and release of 70% of the parasites in the cytosol. One of these mechanisms is the production of acidic substances, such as hemolysin, which has higher activity at more acidic pH levels (5.5), Historical data have shown that during the evolutionary stage of *T. cruzi* the pH of host cells is always below 6.0. In addition, recent studies have shown that the pH of the interior of endosomes and lysosomes ranges between 5.0–6.0 and 4.5–5.0, respectively To prove this fact, the researchers inoculated somewhat more basic solutions (pH 6.2) and measured certain delay in the evolutionary process of the parasite [41–45].

The results obtained in the present work reveal the potential therapeutic innovation because it could be inferred that while the drug remains in the bloodstream—where the pH varies between 7.35 and 7.45—the BNZ@ZIF-8 would be able to release the drug in a controlled and prolonged way. However, when in contact with *T. cruzi* infected cells—whose pH is more acidic (close to 5)—it could be a faster BNZ release. This rapid release can be associated with the ZIF-8 breathing phenomenon, common to some MOFs. Due to the flexible structure ZIF-8 it is possible to observe movements of the organic ligands of the coordination network in acidic pH, which, in turn, open or close the pore windows of the ZIF-8 cavities and enabling the delivery or release of the drug. This phenomenon is best seen in a pH range between 5.0 and 6.0, because at more acidic pHs, ZIF-8 begins to suffer the erosion process that has already been mentioned [46, 47].

Therefore, this phenomenon is of great value to the pharmaceutical sciences when it comes to the development of pH-sensitive DDS since, by modulating the drug release, it would occur only at more acidic pH values, being possible to avoid an intense release at blood pH, thereby avoiding or reducing the incidence of adverse effects and increasing treatment selectivity. However, in vivo studies are still needed to confirm this hypothesis.

Because it is a neglected tropical disease, solid dosage forms are preferred due to easy administration and high compliance treatment. In this case, preformulation studies should consider the enteric coating of capsules or tablets (via polymers, such as Eudragit® or cellulose derivatives, for example) in order to protect them from gastric pH, which would promote a premature release of a large amount of BNZ due to the rapid dissociation of the ZIF-8 framework in a very acidic medium.

## Conclusions

Due to all the presented arguments it was possible to infer that a novel DDS with good incorporation efficiency (IE% = 34%) was obtained through an optimization of an ex situ method. The characterization techniques used in the present study allowed the corroboration of the BNZ@ZIF-8 formation, subsidizing further studies. In vitro dissolution assays under both sink and non-sink conditions evidenced the pH-sensitive drug release and have demonstrated that, at a pH 4.5, the structure of ZIF-8 dissociates rapidly, releasing a large amount of drug in a short time and at pH 7.6 the BNZ release from BNZ@ZIF-8 occurred slowly and with lower burst effect. These data could be corroborated from the AUC values, from which a modulated release is notorious, which could also be proven through model-dependent and -independent methods.

Ultimately, the results mentioned above are of paramount importance to support the preformulation studies for the technological obtaining of innovative solid oral dosage forms aimed at the treatment of Chagas disease.

## Supplementary information

Supplementary Materials
