# Supplementary material for: Benznidazole in vitro dissolution release from a pH-sensitive drug delivery system using Zif-8 as a carrier
Source: J Mater Sci Mater Med. 2021 May 17;32(6):59. doi: 10.1007/s10856-021-06530-w (PMC8128829; doi:10.1007/s10856-021-06530-w)
Supplement: Supplementary file 1 — Supplementary Materials [file 10856_2021_6530_MOESM1_ESM.docx]

*Supplementary Material*

**CHARACTERIZATION OF THE BENZNIDAZOLE:ZIF-8 pH-SENSITIVE DRUG DELIVERY SYSTEM**

Leslie Raphael de Moura Ferraz^a,*^, Alinne Élida Gonçalves Alves Tabosa^a^, Débora Dolores Souza da Silva Nascimento^a^, Aline Silva Ferreira^a^, José Yago Rodrigues Silva^b^, Severino Alves Junior^b^, Larissa Araújo Rolim^c^, Pedro Jose Rolim-Neto^a^.

^a^Laboratório de Tecnologia dos Medicamentos (LTM), Department of Pharmaceutical Sciences, Federal University of Pernambuco, Av. Prof. Arthur de Sá, s/n, Cidade Universitária, 50740-521 Recife - PE, Brazil.

^b^Laboratório de Terras Raras (BSTR), Fundamental Departament of Chemistry, Federal University of Pernambuco, Av. Jornalista Aníbal Fernandes, s/n - Cidade Universitária, 50740-560, Recife-PE, Brazil.

^c^Central Analítica de Fármaco, Medicamentos e Alimentos (CAFMA), Federal University of Vale do São Francisco, Av. José de Sá Maniçoba, s/n, Centro, 56304-917, Petrolina - PE, Brazil.

***Corresponding author:**

E-mail: [prof.raphaelferraz@gmail.com](mailto:prof.raphaelferraz@gmail.com). Tel/Fax (81) 3272-1383. Laboratório de Tecnologia dos Medicamentos (LTM), Department of Pharmaceutical Sciences, Federal University of Pernambuco, Av. Prof. Arthur de Sá, s/n, Cidade Universitária, 50740-521 Recife - PE, Brazil.

**BNZ-ZIF-8 RELEASE STUDY: COMPLEMENTARY CHARACTERIZATION TECHNIQUES**

# Material and methods

## Characterization of BNZ, MF and the BNZ@ZIF-8 by complementary techniques

Different characterization techniques were used to confirm the formation of the system in comparison to the drug alone benznidazole (BNZ) and the physical mixture (MF) between BNZ and ZIF-8.

Spectra of absorption in the Ultraviolet-Visible region (UV-Vis) were obtained by scanning from 190 to 1000 nm. The ultraviolet spectrophotometer SHIMADZU® UV-2401 PC and quartz cuvettes with a cross section of 1 cm were used.

For polarized light microscopy, an Olympus^®^ petrographic microscope, model BX 51, with polarized light was used to identify the optical properties of the samples, using the software of the microscope itself to obtain images within an appropriate scale for recording. A 1 mm thick glass cover was used as a diffuse bottom isotropic medium and as a diffuse background anisotropic medium, a polished hyaline quartz lamina was used, generated through a cut out suballel to the basal section of the crystal and presenting a thickness of 1.1 cm.

Regarding thermal analysis, two techniques were performed: Thermogravimetry (TG) and Differential Exploration Calorimetry (DSC). The TG curves were performed using a Shimadzu® thermocouple, model TGA Q60, under a flowing nitrogen atmosphere of 50 mL.min^-1^, the sample mass being about 3 mg (± 0.05) for BNZ and ZIF-8 isolates and 6 mg (± 0.05) for MFs and BNZ@ZIF-8 in the temperature range of 25 to 1000 °C at the heating rate (β) of 10 °C.min^-1^. Prior to the tests, the thermobalance was checked with calcium oxalate. The BNZ and MF DSC curves were obtained using Shimadzu® DSC-60 Calorimeter, interconnected to the Shimadzu® TA-60WS software, with 50 mL.min^-1^ nitrogen atmosphere and 10 °C.min^-1^ heating rate in the range temperature of 25-500 °C. The samples were placed in a hermetically sealed aluminum sample holder with a mass of 3 mg (± 0,2) for BNZ and ZIF-8 and 6 mg (± 0,2) for MF and BNZ@ZIF-8. The determinations were performed in triplicate and indium and zinc were used to calibrate the temperature scale and the enthalpy response.

The Fourier transform infrared spectroscopy (FTIR) spectra of BNZ, MF and BNZ@ZIF-8 were obtained using the PerkinElmer® equipment (Spectrum 400) with an attenuated total reflectance (ATR) device of selenium crystal. The samples to be analyzed were transferred directly into the ATR device compartment. The results were obtained by scans of 4500 to 600 cm^-1^.

Regarding particle and pore size and volume analyses the samples were dispersed in a 0.02% Triton X 100 surfactant solution and subsequently sonicated for 3 minutes. For the particle size distribution analysis, a Microtac® S3500 particle distribution analyzer was used, with the measurement time of 10 seconds and a flow of 70%. The Fraunhofer method was used. The specific surface area (SBET) was calculated based on the Brunauer-Emmett-Teller method (BET) and the pore size and volume distribution was derived from the Barrett-Joyner-Halenda (BJH) method. Surface area analyzer ASAP2440 Micrometrics® and software for determination of SBET were used. Approximately 200 mg of the BNZ and ZIF-8 samples were degassed for 48 hours at 110 °C for removal of any adsorbed material on the surface of the sample. The analyzes were carried out at the atomization temperature of 160 ºC with air flow of 350 mL/h. The progressive physical adsorption of nitrogen in the material was made at 77K, with subsequent desorption, giving rise to the adsorption/desorption isotherms as suitable models for the adjustment of the experimental points were applied. The BET model was applied in the appropriate portion of the curve according to the value of the surface area (BET and Langmuir).

## In vitro dissolution test under *sink* and *non-sink* conditions

Listed below at the supplementary material table 1 (SM Table 1) are the mathematical equations used for the model-dependent evaluation of the drug release kinetics through dissolution studies.

**SM Table 1** Mathematical models of kinetic study used in the dissolution profile evaluation

| **Model** | **Equation*** | **Graphs plotted** |
| --- | --- | --- |
| **Zero order** | *Q_0_ = Q_t_+ K_0_.t* | *Q_0_ versus t* |
| **First order** | *ln Q_t_ = ln Q_0_ + K_1_.t* | *log Q_t_ versus t* |
| **Higuchi** | *Q_t_ = K_H_.t^1/2^* | *Q_t_ versus t ^½^* |
| **Peppas-Sahlin** | *M_t_/M ͚ = K_1_.t^m^ + K_2_.t^2m^* | *M_t_/M ͚ versus t^m^* |
| **Korsmeyer-Peppas** | *M_t_/M ͚ = K.t^n^* | *M_t_/M ͚ versus t* |

* Q_t_ - amount of drug released at time t; Q_0_ - initial amount of drug in solution; Q ͚ - amount of drug dissolved in the total time; M_t_ - amount of drug dissolved at time t; M ͚ - total amount of drug dissolved when the pharmaceutical form disintegrates completely; K_0_, K_1_, K_2_, K_H_, characteristic constants of each model; t – time

# Results

## Evaluation of drug incorporation in BNZ systems: ZIF-8

**

**

**SM Fig. 1** Scanning of the absorption spectrum in the UV-Vis region of BNZ and ZIF-8

**
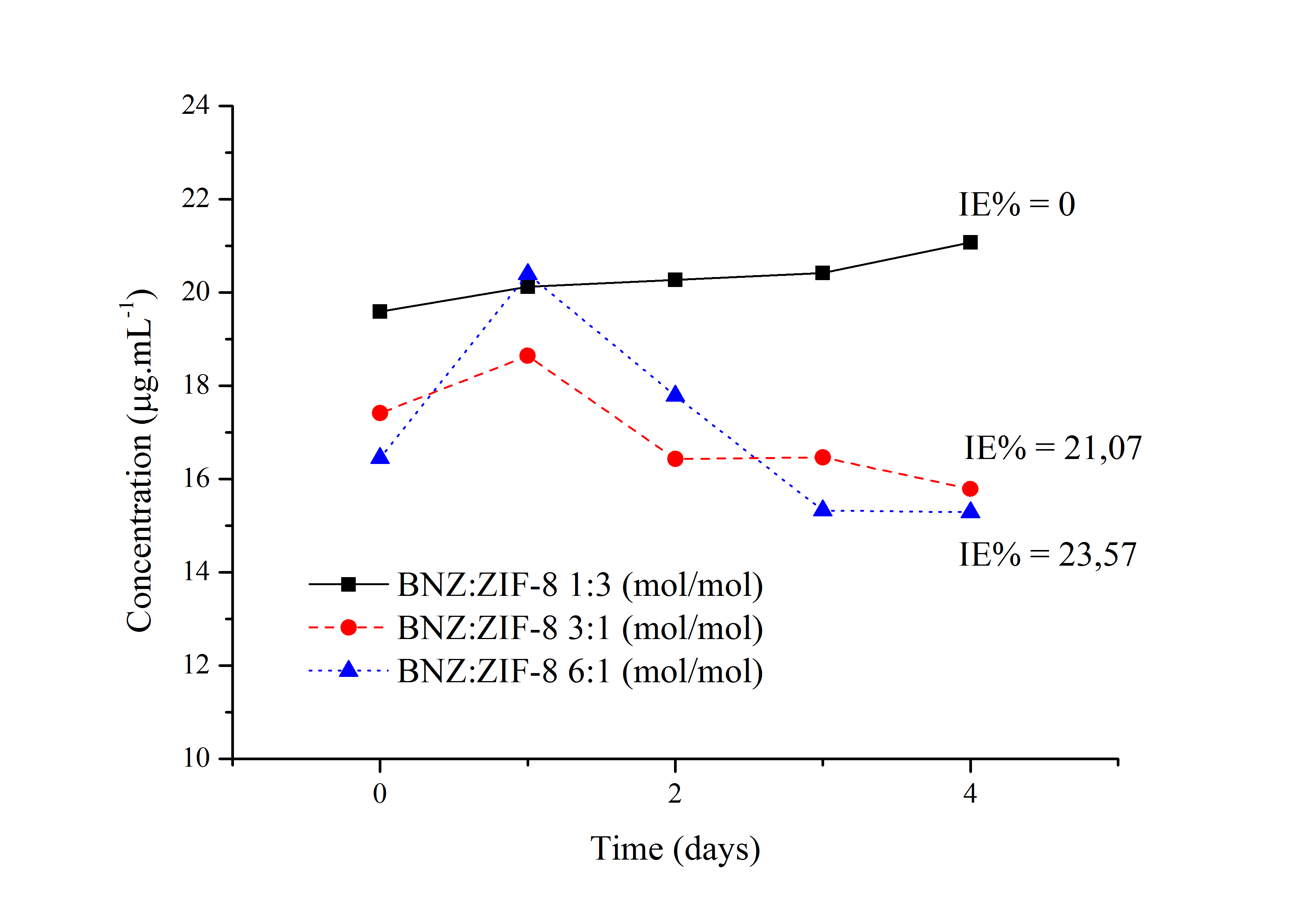
**

**SM Fig. 2** Incorporation curves of BNZ into the ZIF-8 network obtained in acetone with different molar ratios (the dashed line refers to initial concentration, IE% = incorporation efficiency)

**Polarized Light Microscopy**

BNZ was observed in natural light polarized under a transmitted light system and showed transparency and shape of prismatic crystals (SM Fig 3.a). When observed in the orthoscopical illumination system with crossed niches in polarized transmitted light, and placed in an isotropic medium (glass) and the prismatic to circular crystallites, it presented an anisotropic character, revealing the presence of positions of maximum clarity (interference colors) and extinction position (SM Fig 3.b). This behavior is typical of crystalline anisotropic materials [1].

The present work pioneered the characterization of ZIF-8 and derivatives by the MLP technique. The ZIF-8 sample observed in the light-flooded natural light illumination system showed small grains which agglomerate and do not transmit light perfectly. For the analysis of polarized and transmitted light in orthoscopica with crossed nicois, the sample was also submitted to different means: isotropic and anisotropic, represented by glass and quartz, respectively. In both media, the crystals presented an isotropic character and an agglomeration aspect, besides a certain opacity so that the light is not transmitted in its totality (SM Fig 3.c-d). It does not exhibit maximum clearance or extinguishing positions, so that although it is of a crystalline nature, it resembles the behavior of isotropic and amorphous materials.

As expected, the MF presented the anisotropic and isotropic profiles of BNZ and ZIF-8 respectively, corroborating with the results obtained by SEM and showing only the physical adherence of ZIF-8 to the surface of the drug crystals. On the other hand, in the BNZ@ZIF-8 system (SM Fig 3.e-f) it was observed that the anisotropic behavior of the BNZ was little observed (BNZ almost in extinction), which suggests the reduction of crystal size and the formation of more homogeneous systems (SM Fig 3).

**
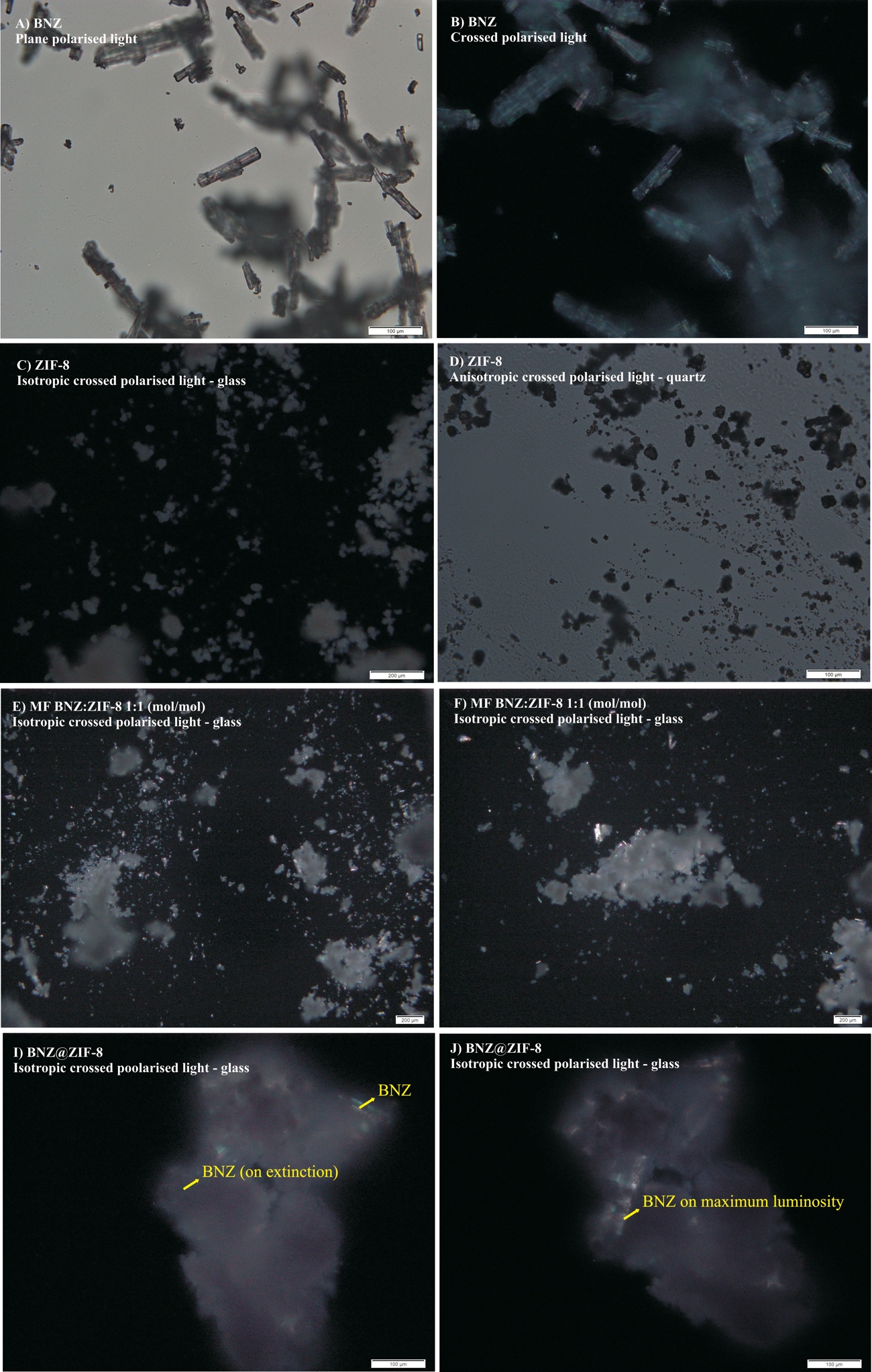
**

**SM Fig. 3** Polarized Light Microscopy of a) and b) BNZ; c) and d) ZIF-8; e) and f) physical mixture; g) and h) BNZ-ZIF-8

**Thermal analysis (DSC, TG/DTG)**

The TG curve of the BNZ (SM Figure 4) showed a mass loss of 1.62%, between 30-105 °C relative to the water content present in the sample. Thermal degradation of the isolated drug was observed in a single event (257.15-297.13 ºC) (DTG peak = 287.43 °C). A mass loss of 45.66% was found.


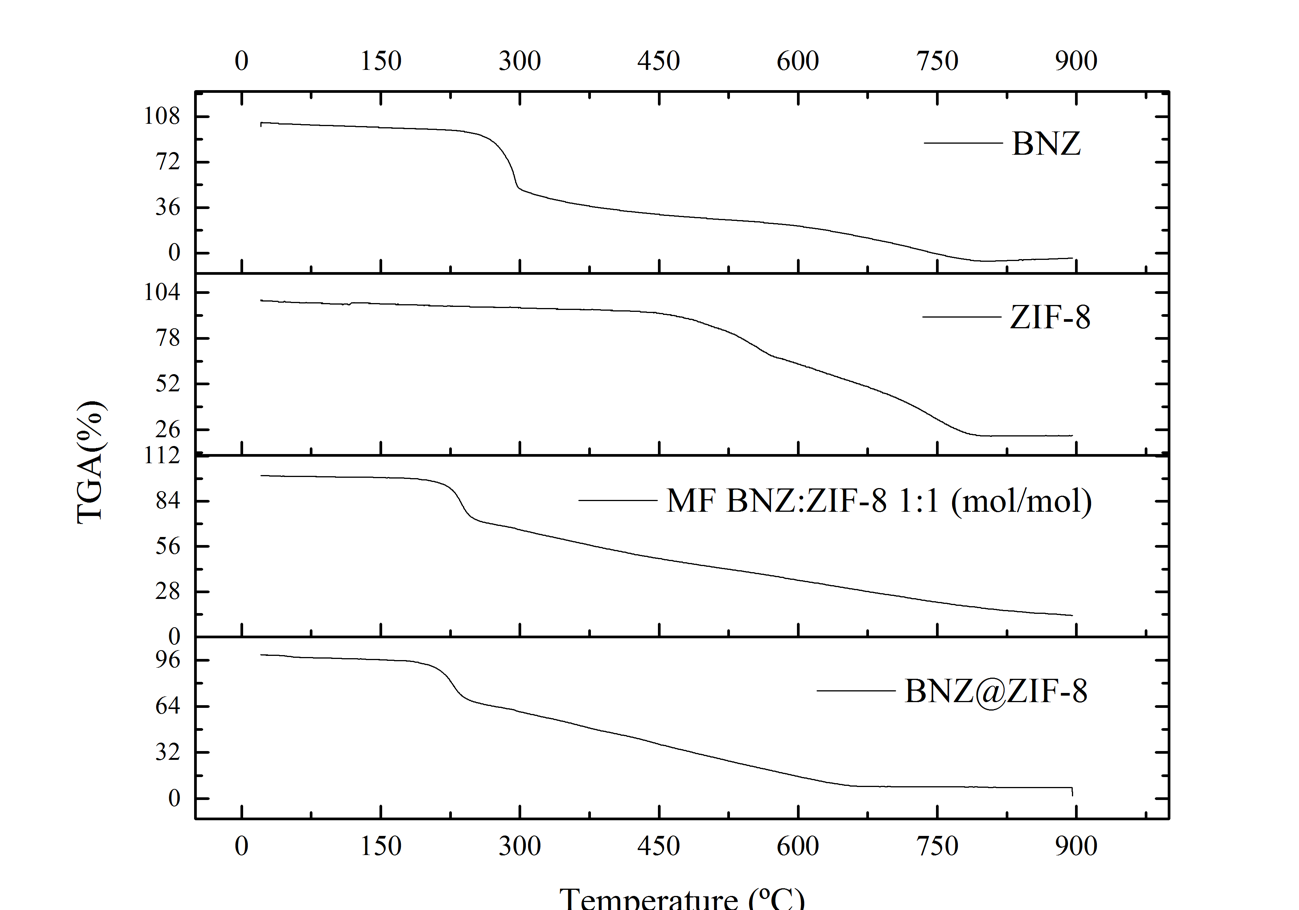


**SM Fig. 4** TG curves of BNZ, ZIF-8, MF and BNZ@ZIF-8 (β=10 ºC.min^-1^)

In the TG curve of ZIF-8 (SM Figure 4), the high thermal stability of the molecule was observed. First, it was possible to show the mass loss of up to 2.16% in the temperature range between 30 and 125 ºC. This value may be related to the loss of water molecules present in the cavities or on the surface of ZIF-8 [2]. At 460.40 °C, there is a loss of only 7% of the initial mass, which evidences the high thermal stability of the ZIF-8, this value can also indicate the exit of water molecules coordinately connected to the ZIF-8 network. In the range of 535.69-578.16 ºC it was possible to observe a significant mass loss – better evidenced by the DTG curve – of 24.32%, related to the molecule organic portion degradation, the imidazolate. Since then, the sample presents slow decay, with low resolution, and no apparent peaks, being difficult to identify them even by the DTG curve, probably caused by thermal decomposition of the inorganic portion of ZIF-8, until the formation of zinc oxide (620.35-720.11 °C - 41.91% mass loss). These results are in line with previous work by important researchers in the field [3-6].

In the TG curve of MF (SM Figure 4) it was possible to show the anticipation of the thermal degradation of the isolated drug, now occurring in the range of 229.89-247.23 ºC (DTGpeak = 238.04 ºC), subsequent to drug melting point. However, a significant reduction of mass loss (24.49%) was observed, suggesting a certain thermal protection. Regarding ZIF-8 degradation, the first event of thermal degradation – related to degradation of imidazolate – was observed in a range quite different from that found for ZIF-8 alone (between 410.89-448.54ºC), also presenting reduction of mass loss (11.55%). This fact suggests that the physical interaction between drug and ZIF-8 may destabilize the organic portion of the latter. Then, the TG curve decayed to approximately 975.07 ºC corresponding to residual zinc oxide (36.78% of mass loss).

In the analysis of the BNZ@ZIF-8 (SM Figure 4), a new mass loss event was observed, between 50.13-87.94 ºC, with a mass loss of 1.97%. Probably, this event is related to the volatilization of acetone that still remains inside the ZIF-8 network, since the decay can be observed from room temperature, with acetone having low vapor pressure and boiling point. This value, smaller than that found for the isolated ZIF-8, still shows that there is a smaller amount of water (moisture) present in the cavity of the ZIF-8 and, therefore, it gives space for the connection with more drug molecules. The rest of the TG curve showed some consonance with the previous results. An anticipation of the drug thermal degradation over the isolated BNZ (223.39-245.34 °C) was also observed [1,4].

However, there was an even more significant reduction in mass loss (26.49%). The degradation of the inorganic portion of ZIF-8 occurred in the range of 321.02-434.88 ° C, with a mass loss of 16.15%; followed by thermal decomposition to zinc oxide (568.51-613.31 °C and 32.78% mass loss). Although it has been shown that ZIF-8 degradation values were anticipated, both showed a significant reduction of the decomposed content. The difference between this thermal profile and that evidenced by MF may be a great indication of the actual formation of the DDS, corroborating the fact that the drug is actually bond with ZIF-8 scaffold [1,4].

The DSC curve of the BNZ (SM Figure 5) showed an intense and defined endothermic peak in the temperature range between 190.04-194.36 °C (T_peak_ = 191.44 °C) (ΔH = 195.52 mJ). Then, the exothermic peak related to drug degradation was observed in the range of 272.57-292.79 °C (T_peak_ = 286.90 °C), which showed large energy release (ΔH = 1.8 J). Similar results were described by Santos and collaborators [7].

The DSC curve of ZIF-8 (SM Figure 5) demonstrates the absence of peaks in the temperature range used, a feature inherent in the nature of the molecule. Since it is an organic-inorganic hybrid molecule, the ZIF-8 degradation events were analyzed through the DTA curve (SM Figure 6), which enabled the sample to heat up to 900 °C. In this, it was possible to observe two endothermic events regarding the phase transition: the first one between 535.69-578.16 ºC; and the second, between 620.35-720.11 °C. These values and their mass losses are described in the discussion of the TG curve of ZIF-8.

Such behavior can be observed in zinc-containing materials. Similar results were discussed by Blachnik and Siethoff [8]. Such solid-solid phase transitions suggest conformational changes of the alkyl chains, where the number and size of the transformations occur as a function of the length and number of the alkyl chains [8].

From the DSC curve of the MF (SM Figure 5) it was possible to evidence the anticipation of the drug melting event (187.82-194.28 ºC). Although this fact can identify interactions between the components of a formulation, the curve also showed the reduction of enthalpy energy related to the event (*ΔH* = 195.52 mJ). This phenomenon is characteristic of polymeric materials, such as: PEG, PVP, HPMC; which makes it possible to increase the solubility of the material, since there is need for a smaller amount of energy to solubilize the drug. Such behavior has already been widely discussed by previous studies of the group [7,9]. Thus, it is clear that simple physical mixing promotes the formation of a positive drug-excipient interaction. On the other hand, there was an intense anticipation of the event related to the drug degradation (T_peak_ = 231.08 ºC), almost occurring subsequent to the melting. However, there was lower energy release (*ΔH* = 1.09 J). These values are in line with those evidenced by the DTA curve.

In the DSC curve of BNZ@ZIF-8 (SM Figure 5), it was possible to observe an endothermic event between 54.17-62.85 ºC, regarding the volatilization of acetone. This behavior, different from that presented in MF, can characterize the formation of the BNZ@ZIF-8. A much more discrete anticipation (184.44-192.91 °C) (T_peak_=189.97 °C) was observed in the drug melting. This variation (approximately 2%) is described by many authors as an acceptable range of compatibility between components of the same formulation [9,10]. However, the value for the enthalpy variation was higher in comparison to the isolated drug (*ΔH* = 261.25 mJ), which may indicate that the system was formed, since a new thermal profile was evidenced. Regarding drug degradation, the exothermic event was anticipated (216.72-244.86 ºC) (T_peak_=234.14 ºC), releasing an energy of 1.8 J, identical to that presented by the isolated drug.

All the above-mentioned information shows the importance of thermal analysis as a technique for the characterization of DDS. Through the TG/DTG, DTA and DSC curves it was possible to corroborate the formation of a DDS, either by changing the thermal profile or attesting compatibility between the used components.


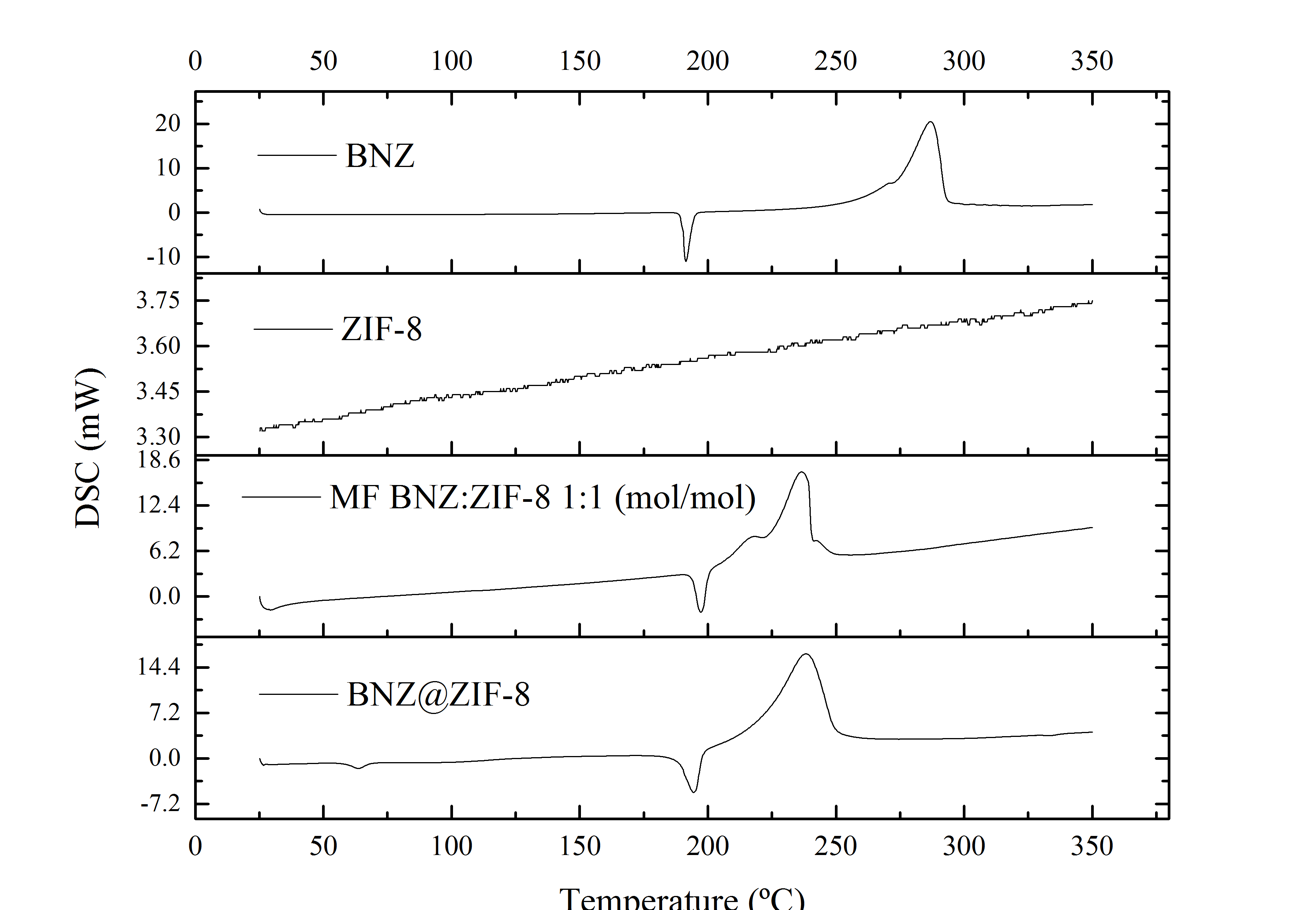


**SM Fig.** 5 DSC curves of BNZ, ZIF-8, MF and BNZ@ZIF-8 (β=10 ºC.min^-1^)

**
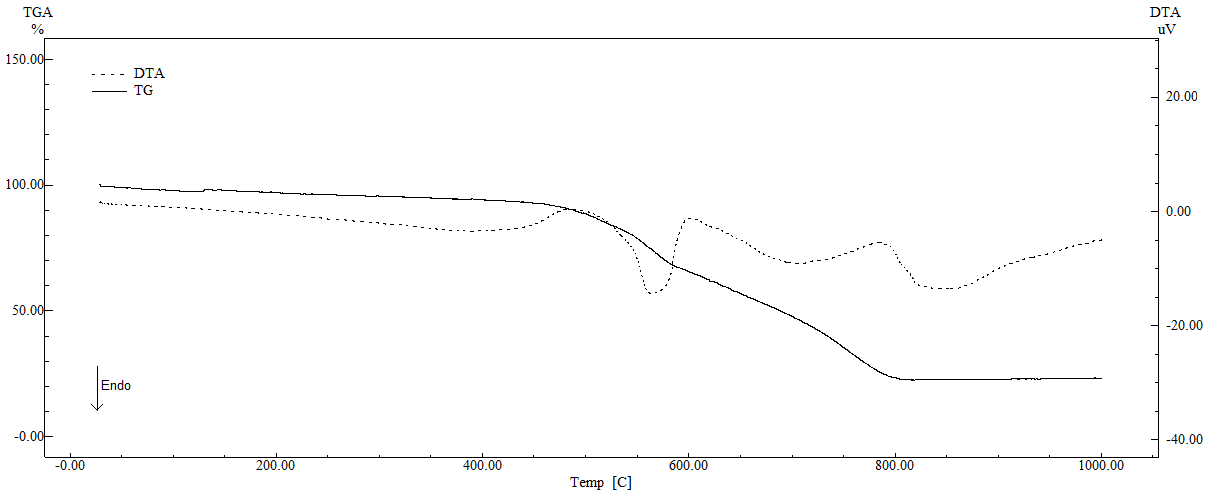
**

**SM Fig. 6** DTA curve of ZIF-8 (β=10 ºC.min^-1^)

**Fourier transform infrared absorption spectroscopy (FTIR)**

The BNZ spectrum (SM Figure 7) shows characteristic peaks, especially when considering typical bands of amides (N-H stretching vibration), carbonyl stretching (amide band I) and N-H deformation (amide band II), in addition to vibrations resulting from the benzyl and imidazole groups, and the nitro group. The band from the N-H stretching vibrations is located at 3266 cm^-1^, the carbonyl stretching band at 1664 cm^-1^ and the NH (amide II) deformation at 1552 cm^-1^, characterizing the secondary amide. In addition, the band at 1292 cm^-1^ is attributed to the C-N stretch. The set of bands at 3033, 3068, 3112 and 3269 cm^-1^ arise from the symmetrical and asymmetric stretching vibrations of the benzene group and the stretching of the aromatic C-H, as shown in SM Figure 7.

The region of the harmonics and combination bands (2000 to 1667 cm^-1^), often useful in determining the number and position of substituents on aromatic rings, appears to be less informative in the spectrum, not being observed the four small bands that would characterize mono-substituted benzene present in the molecule. The band at 1355 cm^-1^ refers to vibration of the symmetrical stretching of the nitro group [11].

From the ZIF-8 spectrum (SM Figure 7) is possible to observe, at 3132, 2962 and 2936 cm^-1^, the presence of C-H aromatic compounds, asymmetric axial deformation and aliphatic C-H stretching relative to the imidazole ring, respectively. In 1670 cm^-1^ a stretch band of C=C was observed, while the absorption C-N bands appeared at 1100 to 1400 cm^-1^ region. It is not possible to observe the behavior of the Zn-N stretch at 450 and 400 cm^-1^, considering that the analyzes were done in equipment that operated up to 600 cm^-1^.

The MF spectrum (SM Figure 7) corresponds to the overlapping of the same bands of BNZ and ZIF-8 when these are analyzed in isolation, it is possible to note the presence of their characteristic peaks. This result suggests a physical interaction only between the drug and ZIF-8 surface due to the sum of the profiles of the isolated materials.

Analyzing the BNZ@ZIF-8 (SM Figure 7), the peaks of the isolated BNZ and ZIF-8 are not well evidenced due to their overlapping. However, it was possible to suggest the presence of the drug in question, due to the presence, in 3266 cm^-1^ and 1292 cm^-1^, of the axial deformation of the N-H and C-N bond, respectively. In 1664 cm^-1^ there is a decrease in the intensity in the carbonyl band, at 1355 cm^-1^ for the nitro group and 1552 cm^-1^ corresponding to the secondary amide. In addition, there was a decrease in the intensity of the absorption band related to the C-N group of ZIF-8 (1400 and 1302 cm^-1^), confirming that it interacts effectively with BNZ [12].

Thus, when comparing the infrared spectra of BNZ@ZIF-8, MF and the isolated substances, it was observed that the system presented the main peaks in a lower intensity, indicating, therefore, the formation of the system due to the signs of interaction between BNZ and ZIF-8 [4,13].

**
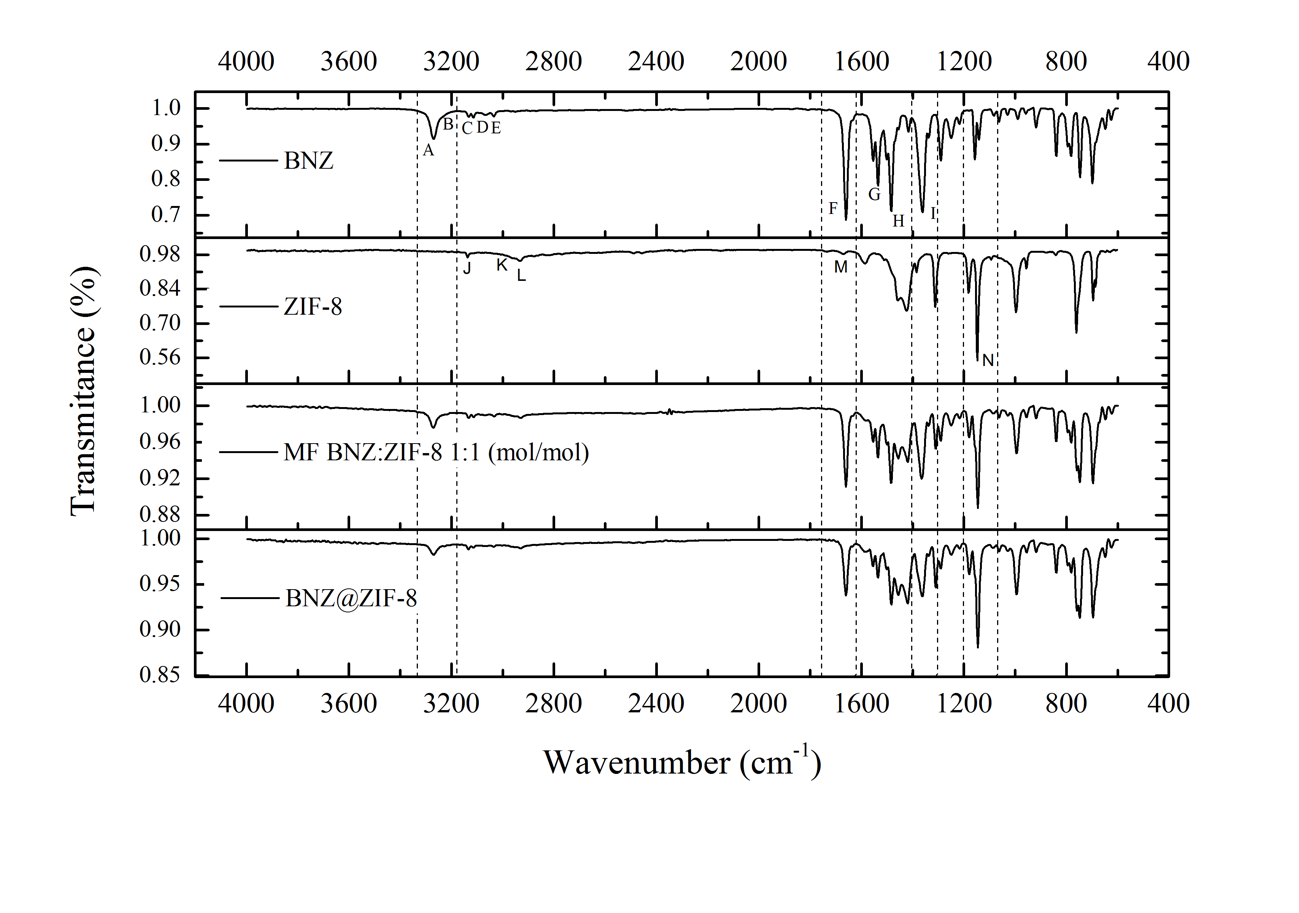
SM Fig. 7** Infrared spectra of BNZ, ZIF-8, MF and BNZ@ZIF-8

# Particle Size by Laser Granulometry and Surface Area Analysis and Pore Size and Volume

The plots of the granulometric distribution of BNZ and ZIF-8 samples are represented by SM Figure 8.a-b. The BNZ crystals had a particle size between 10 and 320 μm, with a mean particle size of 81 μm represented by more than 80% of the sample fraction used in the analysis. A surface area value of 56.18 m^2^.g^-1^ was further elucidated. These values corroborate their slow intrinsic dissolution rate and justify the poor flow properties of the same [14,15].


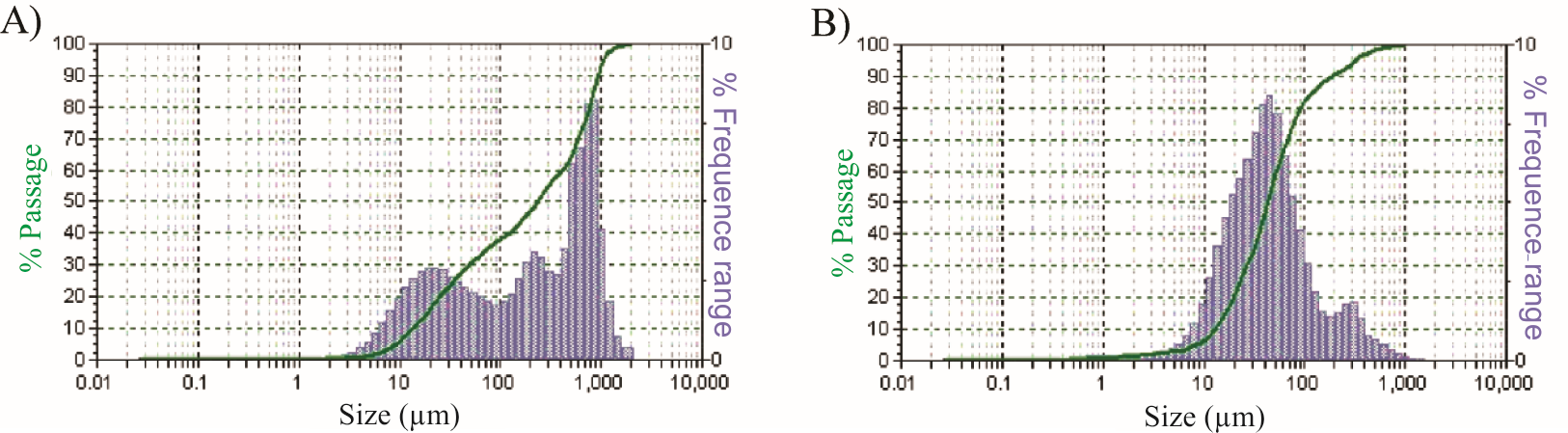
**SM Fig. 8** a) Graph of particle size by laser granulometry of the BNZ and the b) ZIF-8

ZIF-8 presented crystals ranging in size from 13 to 1069 μm, evaluated as a particle aggregate, with a mean particle size of 379 μm (equivalent to more than 80% of the fraction analyzed), with a value of 1386.69 m^2^.g^-1^. These values demonstrate the ability of ZIF-8 to be used as a DDS carrier and are in line with important works in the area [16,17].

The adsorption and desorption curves indicate the type of porosity of the active pharmaceutical ingredient. The BNZ presented a mean pore diameter of 35.42 Å, being an indication of the presence of mesopores (pores with an inner width of 20 and 500 Å). ZIF-8 also demonstrated the presence of mesopores (62.18 Å) [18]. Recent work shows the consonance of these results [16]. However, Fang and collaborators [3] showed the presence of N2 adsorption/desorption isotherms in the ZIF-8 due to the rapid increase in the adsorption volume at low relative pressure, with the mesopores being more predominant.

The MF and the BNZ@ZIF-8 presented, respectively, pore diameters of 42.46 Å and 43.82 Å, maintaining the nature of mesopores, with pore volume increased by 1.065 cm^3^.g^-1^ and 0.602 cm^3^.g^-1^. These results demonstrate the success in obtaining the BNZ@ZIF-8 because the reduction in pore size of the association in relation to the isolated ZIF-8 suggests the presence of the BNZ molecules arranged in the coordination network, interacting with the ZIF-8 and thus reducing its pore size, from 62.18 Å to 43.82 Å [19]. The above data are summarized in SM Table 2.

**SM Table 2** Results of granulometric analysis and surface area, size and pore volume of BNZ and ZIF-8

| **Sample** | **Specific Surface Area (SBET) (m^2^.mL^-1^)** | **Surface Area (BET method) (m^2^.g^-1^)** | **Pore Volume (BJH method) (cm^3^.g^-1^)** | **Pore size**  **(BJH method) (Å)** |
| --- | --- | --- | --- | --- |
| BNZ | 0.30 | 56.18 | 0.050 | 35.42 |
| ZIF-8 | 0.15 | 1386.69 | 0.73 | 62.18 |
| MF |  |  | 1.065 | 42.46 |
| BNZ@ZIF-8 |  |  | 0.602 | 43.82 |

# In vitro release assay

## The following topics present the values related to the study of the drug release kinetics models obtained from dissolution tests in sink and non-sink conditions.

## Dissolution test under sink condition

**SM Table 3** Determination coefficients (R^2^) and R^2^_adjusted_ obtained from fit to kinetic models using the BNZ and BNZ@ZIF-8 system at different pH's (4,5 and 7,6) of the *in vitro* dissolution test under sink conditions

|  | **BNZ** | | **BNZ@ZIF-8** | | **BNZ** | | **BNZ@ZIF-8** | |
| --- | --- | --- | --- | --- | --- | --- | --- | --- |
| **Models** | **pH 4.5** | | | | **pH 7.6** | | | |
|  | R^2^ | R^2^_adjusted_ | R^2^ | R^2^ _adjusted_ | R^2^ | R^2^ _adjusted_ | R^2^ | R^2^ _adjusted_ |
| Zero Order | 0.7649 | 0.7178 | 0.9334 | 0.9200 | 0.6816 | 0.6462 | 0.8544 | 0.8382 |
| First order | 0.9097 | 0.8917 | 0.9953 | 0.9944 | 0.7675 | 0.7417 | 0.9307 | 0.9230 |
| Higuchi | 0.7119 | 0.6543 | 0.9248 | 0.9098 | 0.7489 | 0.7210 | 0.8721 | 0.8578 |
| Korsmeyer-Peppas | 0.9614 | 0.9518 | 0.9647 | 0.9559 | 0.9394 | 0.9243 | 0.9081 | 0.8851 |
| Peppas-Sahlin | 0.9125 | 0.8542 | 0.9930 | 0.9883 | 0.7489 | 0.5816 | 0.9810 | 0.9683 |

**SM Table 4** Release constants and coefficients obtained from fitting to kinetic models using BNZ@ZIF-8 system at different pH's in sink conditions

| **Models** | **pH 4.5** | | **pH 7.6** | |
| --- | --- | --- | --- | --- |
|  | **K_1_** | **K_2_** | **K_1_** | **K_2_** |
| Peppas-Sahlin | 1.78 | -1.14 | 1.41 | 1.14 |
|  | **N** | | **n** | |
| Korsmeyer-Peppas | 1.86 | | 1.25 | |

**SM Table 5** Similarity factor values ƒ2 comparing *in vitro* dissolution profiles in sink conditions of BNZ-MF and BNZ-BNZ@ZIF-8 in pH's 4,5 and 7,6

| **Comparative dissolution profile** | **ƒ2** | |
| --- | --- | --- |
|  | **pH 4.5** | **pH 7.6** |
| BNZ-MF | 37.05 | 43.78 |
| BNZ-BNZ@ZIF-8 | 46.12 | 45.42 |

## Dissolution test under non-sink condition

**SM Table 6** Determination coefficients (R^2^) and R^2^_adjusted_ obtained from the adjustment to kinetic models using the BNZ and BNZ@ZIF-8 system at different pH's (4,5 and 7,6) at *in vitro* non-sink conditions dissolution test

|  | **BNZ** | | **BNZ@ZIF-8** | | **BNZ** | | **BNZ@ZIF-8** | |
| --- | --- | --- | --- | --- | --- | --- | --- | --- |
| **Models** | **pH 4.5** | | | | **pH 7.6** | | | |
|  | R^2^ | R^2^_adjusted_ | R^2^ | R^2^ _adjusted_ | R^2^ | R^2^ _adjusted_ | R^2^ | R^2^ _adjusted_ |
| Zero order | 0.6641 | 0.6221 | 0.6249 | 0.5780 | 0.5049 | 0.4599 | 0.7447 | 0.7214 |
| First order | 0.8748 | 0.8592 | 0.7601 | 0.7301 | 0.7089 | 0.6824 | 0.9004 | 0.8913 |
| Higuchi | 0.6463 | 0.6021 | 0.7992 | 0.7741 | 0.7119 | 0.6857 | 0.8491 | 0.8354 |
| Korsmeyer-Peppas | 0.8626 | 0.8454 | 0.9915 | 0.9904 | 0.9614 | 0.9579 | 0.8956 | 0.8862 |
| Peppas-Sahlin | 0.9997 | 0.9996 | 0.9842 | 0.9797 | 0.9125 | 0.9045 | 0.9896 | 0.9875 |

**SM Table 7** Release constants and coefficients obtained from adjustment to kinetic models using BNZ@ZIF-8 system at different pH's in non-sink conditions

| **Models** | **pH 4.5** | | **pH 7.6** | |
| --- | --- | --- | --- | --- |
|  | **K_1_** | **K_2_** | **K_1_** | **K_2_** |
| **Peppas-Sahlin** | 1.89 | -1.92 | 0.72 | -2.59 |
|  | ***n*** | | ***n*** | |
| **Korsmeyer-Peppas** | 1.64 | | 0.91 | |

**SM Table 8** Similarity factor values ƒ2 comparing BNZ-MF and BNZ-BNZ@ZIF-8 at pH's 4.5 and 7.6 under non-sink conditions.

| **Comparative dissolution profile** | **ƒ2** | |
| --- | --- | --- |
|  | **pH 4.5** | **pH 7.6** |
| BNZ-MF | 52.42 | 44.41 |
| BNZ-BNZ@ZIF-8 | 51.20 | 36.78 |

**References**

[1] Weaver R. Rediscovering polarized light microscopy. Am. Lab. 2003;35:55–61.

[2] Blachnik R, Siethoff C. Thermoanalytical and X-ray study of some alkylammonium tetrachlorozincates. Thermochim Acta. 1996;278:39–47. https://doi.org/10.1016/0040-6031(95)02768-8.

[3] Fang M, Wu C, Yang Z, Wang T, Xia Y, Li J. ZIF-8/PDMS mixed matrix membranes for propane/nitrogen mixture separation: Experimental result and permeation model validation. J. Memb. Sci. 2015;474:103-113.

[4] Rodrigues MO, Paula MV, Wanderley KA, Vasconcelos IB, Alves S, Soares T. Metal organic frameworks for drug delivery and environmental remediation: A molecular docking approach. Int. J. Quantum Chem. 2012;112:3346–3355.

[5] Liu S, Xiang Z, Hu Z, Zheng X, Cao D. Zeolitic imidazolate framework-8 as a luminescent material for the sensing of metal ions and small molecules. J. Mater. Chem. 2011;21:6649–6653.

[6] Ordoñez MJC, Balkus KJ, Ferraris JP, Musselman IH. Molecular sieving realized with ZIF-8/Matrimid^®^ mixed-matrix membranes. J. Memb. Sci. 2010;361:28–37.

[7] Santos FL, Rolim LA, Figueirêdo CB, Lyra MA, Peixoto MS, Ferraz LR, Soares-Sobrinho JL, Lima AAN, Leite ACL, Rolim-Neto PJ. A study of photostability and compatibility of the anti-chagas drug Benznidazole with pharmaceutics excipients. Drug Dev Ind Pharm. 2015;41:63-69.

[8] Blachnik R, Siethoff C. Thermoanalytical and X-ray study of some alkylammonium tetrachlorozincates. Thermochim Acta. 1996;278:39–47. <https://doi.org/10.1016/0040-6031(95)02768-8>.

[9] Costa SPM, Silva KER, Medeiros GCR, Rolim LA, Oliveira JF, Lima MCA, Galdino SL, Pitta IR, Rolim-Neto PJ. Thermal behavior and compatibility analysis of the new chemical entity LPSF/FZ4. Thermochim. Acta. 2013;562:29–34.

[10] Melo CM, Vieira ACQM, Nascimento ALS, Figueirêdo CBM, Rolim LA, Soares-Sobrinho JL, Veras LMC, Leite JRSA, Rolim-Neto PJ, Soares MFLR. A compatibility study of the prototype epiisopiloturine and pharmaceutical excipients aiming at the attainment of solid pharmaceutical forms. J. Therm. Anal. Calorim. 2014;120:689–697.

[11] Rolim LA. Estudo de Degradação Do Fármaco Benznidazol Utilizado No Combate a Doença de Chagas Por Hidrólise, Oxidação, Fotólise e Termodegradação [Dissertação]. Recife: Universidade Federal de Pernambuco; 2010.

[12] Vasconcelos IB, Silva TG, Militão GCG, Soares TA, Rodrigues NM, Rodrigues MO, Costa NB, Freire RO, Alves-Junior S. Cytotoxicity and slow release of the anti-cancer drug doxorubicin from ZIF-8. RSC Adv. 2012;2:9437-9442.

[13] Gomes TA, Costa SPM, Medeiros GCR, Silva CR, Lyra MAM, Silva KER, Albuquerque MM, Silva RMF, Rolim-Neto PJ. Estratégias utilizadas para o incremento da solubilidade do fármaco antiretroviral classe II : Efavirenz. J. Basic Appl. Pharm. Sci. 2015;36:239–249.

[14] Shekunov BY, Chattopadhyay P, Tong HHY, Chow AHL. Particle Size Analysis in Pharmaceutics: Principles, Methods and Applications. Pharm. Res. 2007;24:203–227.

[15] Rhodes M. Introduction to Particle Technology, second ed. [S.I.]: Wiley; 2008.

[16] Hu X, Yan X, Zhou M, Komarneni S. One-step synthesis of nanostructured mesoporous ZIF-8 / silica composites. Micro Meso Mat. 2016;219:311-316.

[17] Sun DD, Ju TCR, Lee PI. Enhanced kinetic solubility profiles of indomethacin amorphous solid dispersions in poly(2-hydroxyethyl methacrylate) hydrogels. Eur. J. Pharm. Biopharm. 2012;81:149-158.

[18] Webb PA, Orr C. Analytical Methods in Fine Particle Technology, first ed. New York: Micromeritics; 1997.

[19] Bidone J, Melo APP, Bazzo GC, Carmignan F, Soldi MS, Pires ATN, Lemos-Senna E. Preparation and characterization of ibuprofen-loaded microspheres consisting of poly(3-hydroxybutyrate) and methoxy poly (ethylene glycol)-b-poly (D,L-lactide) blends or poly(3-hydroxybutyrate) and gelatin composites for controlled drug release. Materials Science and Engineering. 2009;29:588–593.
